# An Update of Tetrodotoxins Toxicity and Risk Assessment Associated to Contaminated Seafood Consumption in Europe: A Systematic Review

**DOI:** 10.3390/toxins17020076

**Published:** 2025-02-08

**Authors:** Carlo Varini, Maura Manganelli, Simona Scardala, Pietro Antonelli, Carmen Losasso, Emanuela Testai

**Affiliations:** 1 Istituto Superiore di Sanità, Department of Environment and Health, 00161 Rome, Italy; carlo.varini@iss.it (C.V.); maura.manganelli@iss.it (M.M.); simona.scardala@iss.it (S.S.); 2 Istituto Zooprofilattico Sperimentale delle Venezie, Microbial Ecology and Microorganisms Genomics Laboratory, 35020 Legnaro, ItalyCLosasso@izsvenezie.it (C.L.); 3 National PhD Programme in One Health Approaches to Infectious Diseases and Life Science Research, Department of Public Health, Experimental and Forensic Medicine, University of Pavia, 27100 Pavia, Italy

**Keywords:** TTX, toxicity, mollusks, gastropods, pufferfish, risk assessment

## Abstract

Following the occurrence of Tetrodotoxins (TTXs) in Europe—a group of neurotoxins identified in Asia, where fatalities occurred after the ingestion of contaminated pufferfish—the EFSA proposed a limit of 44 µg of TTX/kg of shellfish meat in mollusks in 2017, to protect heavy consumers. The limit was based on an acute reference dose (ARfD) derived from the few available data on TTX toxicity. TTX is expected to increase with sea-surface warming; indeed, it has been found in spring/summer in mollusks in Europe, with concentrations often exceeding this limit. Due to the numerous uncertainties of the EFSA’s ARfD, we conducted a systematic review to provide an update on TTX toxicity. Out of 12,741 articles retrieved from PubMed, Science Direct, and Scopus since 2017, only 17 were eligible for data extraction. Our results show that they are not sufficient to modify the EFSA’s conclusions. Furthermore, our analysis of occurrence data in European seafood, to assess the current risk of exposure to TTX, reveals several gaps, such as different LODs/LOQs and seasonal monitoring not allowing comparisons between areas and too few analyzed sites. However, the presence of positive samples exceeding the EFSA limit indicates a potential risk even for general consumers, highlighting the urgency to address these knowledge gaps.

## 1. Introduction

In the European Union (EU), the safety of seafood (including fish, mollusks, and few other invertebrates) is strictly controlled with respect to a number of chemical compounds, including various marine biotoxins, whose occurrence is monitored in live bivalve mollusks [1,2,3]. The routine monitoring program in place for Paralytic Shellfish Toxins (PSTs), Diarrheic Shellfish Toxins, Yessotoxins (YTXs), Amnesic Shellfish Toxins (ASTs), and Azaspiracids (AZAs) aims to ensure that legal limits are not exceeded, thereby avoiding contaminated seafood reaching consumers and protecting their health. However, in recent years, other groups of marine biotoxins, including Tetrodotoxin (TTX) and its analogs, have emerged in the Mediterranean Sea and European Atlantic Ocean [4,5]. TTXs are a group of potent neurotoxins named after the Tetraodontidae fish family, in which they were first characterized [6]. Until a few years ago, the presence of TTXs had been predominantly reported in Asia within the Pacific Ocean area, where the contamination of pufferfish, consumed as a delicacy, and gastropods had caused several severe cases of poisoning in Japan and China, some of which resulted in fatalities [7,8]. To explain the appearance of TTXs in European seas, the influx of biota from the Red Sea to the Mediterranean through the Suez Canal was initially suggested [9]. However, more recent analyses suggest that the proliferation of toxin-producing organisms is favored by the warming of sea waters, likely due to climate changes [10,11]. Indeed, the modification of parameters such as temperature, dissolved oxygen, and water acidity, as well as the increasing concentration of nutrients and eutrophying compounds (due to increasingly frequent flood or drought events), can lead to massive growths in microalgae and/or enable species typically found in tropical areas to migrate to other regions, including temperate European waters [10,12,13]. Natural toxins as a category and specifically TTXs are considered among the emerging issues related to climate change, as shown by the results from the CLEFSA (CLimate change and Emerging risks for Food SAfety) project [14].

TTXs have not yet been included among the list of regulated marine biotoxins in live bivalve mollusks. This is probably because, so far in Europe, the only human intoxication case report was described by Rodríguez et al. [15], where a person was hospitalized following the ingestion of a TTX-contaminated gastropod species, the trumpet shell *Charonia lampas lampas*. After that event, TTX was detected in mollusks in England [4] and Greece [5]. As evidence of TTX occurrence in European seafood accumulated, the European commission requested an opinion on TTX risk for European mollusk consumers [16]. The concern was justified since TTX has been reported to have the highest mortality rate among all marine biotoxin poisonings [17].

Several uncertainties and gaps were identified in the risk assessment, which was limited to acute effects; by applying a highly conservative approach, an acute reference dose (ARfD) of 0.25 μg/kg bw was proposed. Considering an average adult weighing 70 kg and the consumption of a 400 g portion of seafood [18] (to protect the health of heavy consumers), the EFSA established a limit of 44 μg/kg of seafood for the presence of TTX in gastropods and marine bivalves intended for human consumption. This value is lower than the limit value set in Japan and China, where the TTX concentration in pufferfish flesh considered not to cause adverse effects is around 2 mg/kg [7,19].

So far, only the Netherlands and France have adopted the EFSA recommendation within their national plans for the monitoring of TTXs in live bivalve mollusks [20,21], while no harmonized legislation has been adopted within the EU [22]. Concerns have also been raised about the conservativism of the limit [22], as well as the possibility of jeopardizing the shellfish industry’s viability [23,24], with frequent shellfish area closures. In order to obtain more information, in its opinion, the EFSA recommended the Member States to gather more information on TTX occurrence in bivalves harvested in EU waters [16]. Since 2017, various reports have been published, indicating that TTX contamination is widespread across Europe [20,22,25,26,27,28,29,30,31,32,33,34,35,36].

Italy is characterized by a large area devoted to shellfish farming [37], being the second largest producer of Mediterranean mussels (*Mytilus galloprovincialis*) with 61,921.4 tons and the largest for Manila clams (*Ruditapes philippinarum*) with 23,053.2 tons in 2022 [38]. Since TTX has been detected, so far, in few examined farming areas [22], exposure to TTXs via seafood consumption can represent a possible emerging concern for human health, also considering the consolidated Italian tradition of consuming shellfish delicacies.

With the aim of gathering more information on TTX contamination in Italy, the Ministry of Health has recently funded a dedicated project titled “Risk Mitigation Strategies and Tools for an Ongoing Problem: Tetrodotoxins (TTXs), a Group of Emerging Toxins in Live Bivalve Mollusks Intended for Human Consumption”, coordinated by the Istituto Zooprofilattico Sperimentale delle Venezie (IZSVe). The project employs an integrated approach that combines early warning systems, innovative diagnostic tools, and risk assessment methods with the primary goal of understanding the phenomenon of TTX contamination in bivalve mollusks intended for human consumption in Italy and the associated potential risk to propose mitigation measures. The study described in this paper is part of this project, with the aim of the following: (i) updating the TTX hazard characterization based on new data published after the publication of the EFSA opinion [16], by using a systematic review approach, and (ii) collecting new occurrence data from the available literature and European seafood consumption data from the EFSA FoodEX2 database, in order to carry out an updated risk assessment for European countries.

### 1.1. Tetrodotoxin: General Information

**Figure 1:**
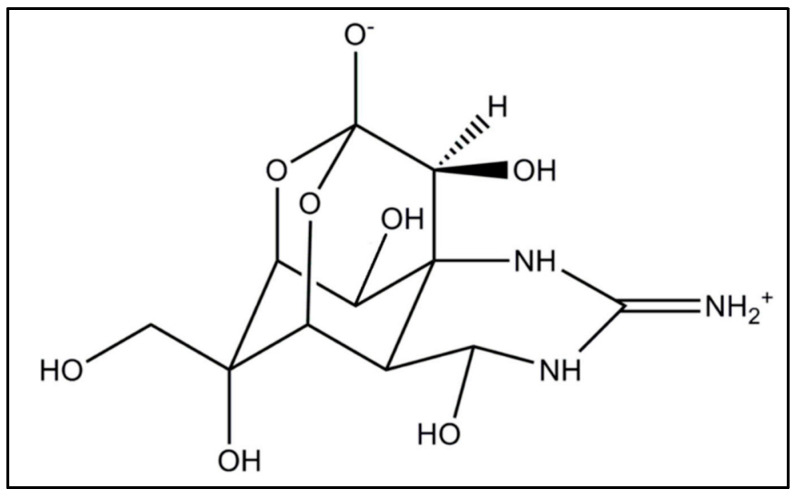
Molecular structure of Tetrodotoxin.

Tetrodotoxin (Figure 1) is a highly hydrophilic crystalline solid (MW is 319.27 g/mol) with a LogP of −5.9 that enables solubility, besides in water, in diluted acetic acid, diluted alcohol, and ether, but it is almost insoluble in any other organic solvent. For this reason, bioaccumulation in adipose tissue is highly unlikely.

TTX is a heat-stable compound that remains stable at boiling temperatures, making it resistant to destruction through cooking [39], except in the presence of strongly acidic or alkaline solutions. It displays an acid–base behavior with a dissociation constant, pK_a_, of 8.76; therefore, in physiological conditions, the toxin exists as a cation in the NH_3_ group (https://pubchem.ncbi.nlm.nih.gov/compound/11174599 (accessed on 15 January 2024)).

At least 25 TTX analogs are known to date, which can be classified into four groups: (i) analogs that are chemically equivalent to TTX (e.g., 4-epiTTX and 4,9-anhydroTTX), (ii) deoxy analogs (e.g., 5-deoxyTTX, 11-deoxyTTX, 6,11-dideoxyTTX, and 5,6,11-trideoxyTTX), (iii) 11-CH_2_OH-oxidized analogs (e.g., 11-oxoTTX), and (iv) analogs lacking C11 (e.g., 11-norTTX-6(S)-ol and 11-norTTX-6(R)-ol) [16]. The origin of TTX contamination in marine environments is still under investigation, with various hypotheses proposed. It is well known that TTXs are produced by different bacterial strains isolated from TTX-bearing organisms, including marine species [40]. Several bacterial genera, such as *Vibrio*, *Pseudoalteromonas*, and *Shewanella*, have been isolated from TTX-harboring organisms like pufferfish, pointing to a microbial synthesis route for TTXs. However, in vitro studies aimed at culturing these strains and stimulating TTX production are arduous, often requiring years to achieve the expected results [41]. Regarding bivalves, some authors speculate about the involvement of marine phytoplankton species such as *Alexandrium tamarense* and *Prorocentrum cordatum* [5,42], both potentially toxic [43,44]. Although *P. cordatum* presence in water columns correlates with higher TTX levels in bivalves collected from the same region, suggesting a potential ecological link [5,25], the production of TTXs in the dinoflagellate species has never been confirmed. A further possibility is the presence of TTX-producing bacterial species living in a mutualistic relationship with phytoplankton. In recent analyses, bacterial taxa such as *Shewanella* sp., members of *Vibrionaceae*, and *Flavobacteriaceae* have been detected in TTX-positive bivalve flesh, often alongside *P. cordatum*. This co-occurrence hints at a microbial contribution to TTX levels influenced by environmental factors [25]. This finding is corroborated by recent evidence showing that TTXs in live bivalve mollusks occur generally at temperatures between 15 and 20 °C [30]. Finally, the role of TTX-bearing fishes such as *Lagocephalus sceleratus* is still debated. This pufferfish species, originating from warmer latitudes, has expanded its range across the Mediterranean basin [24], likely entering through the Suez Canal as a Lessepsian migrant. TTXs may accumulate in marine food webs [45], including in *L. sceleratus*, with marine bivalves stockpiling the toxin in their flesh due to their filter-feeding habits. This trophic transfer mechanism could help explain the presence of TTXs in some shellfish species, emphasizing the complex ecological web contributing to TTX exposure in marine environments.

### 1.2. Effects of TTX

TTX acts by blocking the voltage-gated sodium channels of neuromuscular cells [46]; therefore, its mechanism of action and symptoms are similar to those of saxitoxins (STXs) [47,48]. The intoxication, generally associated with the ingestion of contaminated seafood, results in a series of acute symptoms, starting from perioral numbness and paresthesia, lingual numbness, early motor paralysis, incoordination, slurred speech, vomiting, headache, dizziness, muscle weakness, and ataxia and progressing to paralysis of the respiratory muscles, which leads to respiratory (or cardiac) failure and, ultimately, death. Death is primarily caused by respiratory failure resulting from the blockage of the phrenic nerve, which prevents thoracic expansion. Poisoning from TTX is clinically graded on a scale from 1 to 4, created in 1941 by Fukuda and Tani [49], that focuses on the severity of the symptoms, from mild to severe, life-threatening situations. Symptoms, usually, appear 10–45 min after ingestion with a possible delayed response 3–6 h after initial contact with a dose–response trend. Cardiac effects are registered only at high doses with clinical signs of hypotension, bradycardia, and cardiac arrhythmia [47,50].

The diagnosis of TTX intoxication is typically based on clinical history (anamnesis) and the interpretation of symptoms [7,16]. The direct analysis by HPLC MS/MS of body fluids (e.g., urine or blood) and/or unconsumed residual food may confirm the presence of TTX, although these samples are rarely available [7,16].

Since there are no available antidotes or specific pharmacological treatments, in cases of poisoning, only symptomatic treatment and respiratory support (mechanical ventilation) can be provided, with varying recovery times, depending on the total elimination of the toxins from the organism [47,50].

Human exposure to TTX occurs mainly via food consumption, but its toxicokinetic behavior after ingestion is not yet well understood. The very little available information is mainly based on human data recorded during intoxication events [16]. The rapid onset (starting from 10 min) of adverse effects seems to suggest fast gastrointestinal absorption, but no information on its distribution is available. TTX can be tracked in blood from 6 up to 24 h, depending on the concentrations ingested. In critical cases, TTX has been found several days after recovery. Data gaps can be identified regarding the biotransformation, metabolite identification, main detoxification pathways, and burden of each excretion route.

All adverse effects are linked to the inhibition of sodium ion channels called Na_v_ channels, for which TTX shows a very high affinity [51]. Na_v_ channels are a family of voltage-operated channels responsible for ion flow across cellular membranes, playing a critical role in the generation and propagation of action potentials in neuronal cells of both the central and peripheral nervous system [52,53]. The direct interaction of TTX with Na_v_ channels disrupts action potential generation and propagation, leading to altered neuronal excitability [54]. Among the nine known Na_v_ isoforms (1.1–1.9), TTX strongly interacts with the Na_v_ isoforms 1.1, 1.2, 1.3, 1.4, 1.6, and 1.7 [51,55], with an inhibition concentration below 10 nM. Na_v_ 1.1, Na_v_ 1.2, Na_v_ 1.3, Na_v_ 1.6, and Na_v_ 1.7 are located in the central and peripheral nervous system [53]. However, TTX’s effects are largely restricted to the peripheral nervous system (PNS) due to its hydrophilic nature, which makes it unlikely to cross the blood–brain barrier (BBB) [56]. Na_v_ 1.4, abundant in skeletal muscles, accounts for the paralytic action of the toxin [55,57,58]. TTX interacts with Na_v_ 1.5, which is more prominent in the cardiac tissue, only at extremely high doses (IC50 in the 1.5–2.5 µM range [51]), so effects on the heart are reported only after the ingestion of very high concentrations.

From a molecular point of view, TTX’s guanidine group, adopting a lateral orientation, is able to interact, by a network of hydrogen bonds, with three acidic residues on the outer side of the selectivity filter of the sodium channel pore [59]. Structural analogs of TTX, such as 4,9-AnhydroTTX and 5,6,11-DeoxyTTX, have fewer available oxygen groups, resulting in weaker binding and lower stability in the toxin–receptor complex, making them less toxic than TTX.

## 2. Methodology

### 2.1. Searching Strategies

A systematic review of the primary scientific literature was completed according to the EFSA guidance [60] and PRISMA guidelines (https://www.prisma-statement.org/prisma-2020. Last accessed on 23 January 2025) to gather all the available information regarding the potential health effects of TTX to re-evaluate the hazard characterization 6 years after the EFSA opinion. Data on occurrence in Europe in seafood products intended for the market or directly consumed by fishermen and their families (to update data on TTX occurrence in mollusks and other seafood in Europe) and data on the occurrence of TTXs in the invasive alien species of pufferfish in the Mediterranean Sea were collected following the same criteria. Pufferfishes are now easily found in the Mediterranean Sea, and even if they are not traditionally eaten in Europe, they could be eaten accidentally by fishermen or immigrants coming from Asiatic countries.

This research was performed using three different database platforms: Scopus, PubMed, and Science Direct. For each search run, the searching strategies, including the search terms used for the different databases, day, and time were annotated and reported (Table S1) to grant transparency to the process as well as reproducibility. The search terms were chosen to include as much as possible studies relevant to the scope and to minimize bias.

All the articles retrieved were saved and imported into the EndNote^TM^ online platform.

### 2.2. Selection of Papers

#### 2.2.1. Primary Screening

After duplication and out-of-scope article removal, the screening of the titles and abstracts of relevant papers was based on the following exclusion criteria:

- A language different from English.
- A congress abstract (whose quality could not be checked).
- Publication date. Hazard characterization papers published before 2016 were already included in the EFSA opinion [16]. Occasionally, some older studies were retrieved as the base of forward snowballing.

Reviews were used for consultation (not being a primary literature source) as well as for snowballing. Articles that did not meet the inclusion criteria were excluded from further analysis. The full text of each article that passed the primary screening was retrieved for secondary screening.

For data on occurrence, papers published before 2021, the time of the last comprehensive review [22], were excluded. No time frame was set for occurrence in pufferfish.

#### 2.2.2. Secondary Screening and Data Extraction

The methodological quality of the selected articles was evaluated using the full-text articles. Aspects such as the design, execution, analysis, and reporting of the studies, which may have led to biased results, were considered. The selection of articles for data extraction was performed on the basis of expert judgment by one operator and, in the case of doubt, after discussion with one or two other experts, who nevertheless randomly re-examined a small subsample of the literature under review. Articles selected for hazard characterization were evaluated for their reliability, relevance, and adequacy on the basis of the Klimisch score [61] by using the ToxRTool (a tool to assess the reliability of toxicological data), a spreadsheet developed by ECVAM (https://joint-research-centre.ec.europa.eu/scientific-tools-and-databases/toxrtool-toxicological-data-reliability-assessment-tool_en (accessed on 10 September 2023)) [62]. Each record selected for data extraction was screened independently by two researchers, and since no striking discrepancy on the outcome of the ToxRTool occurred, it was not necessary to involve a third expert. All the data considered useful for hazard characterization were captured in a preliminarily defined excel data extraction form, and the certainty assessment was performed on the basis of expert judgment.

For occurrence, records providing an adequate description of the analytical method used were included [63]. The EFSA’s assessment of TTX detection and quantification methods [16] was used as a basis for evaluating the studies. Studies with results obtained through an ineffective quantification method (which did not provide LOD and LOQ values, did not take into account potential matrix effects, and did not provide recovery information) were not considered for the quantification of occurrence.

### 2.3. Exposure and Risk Assessment

To assess the risk for human health of TTX in Europe, following the internationally accepted procedure [64], exposure was calculated by combining data on its occurrence in seafood, retrieved from an extensive literature search (ELS), with data on consumption taken from FoodEx2 (https://www.efsa.europa.eu/en/microstrategy/foodex2-level-7 (accessed on 15 December 2024)), the EFSA consumer database available online for consultation. An excel worksheet was obtained for acute exposure, using the acute food consumption data, which were selected from the most recent surveys applying adequate filters at each hierarchy level (7 levels) with the removal of unwanted information. Once data were downloaded from the EFSA website and put into an excel worksheet, only statistically significant 95th percentile or, when not available, significant average were used, based on the number of consuming days.

## 3. Results and Discussion

### 3.1. Bibliographic Search

#### 3.1.1. Hazard Characterization

**Figure 2:**
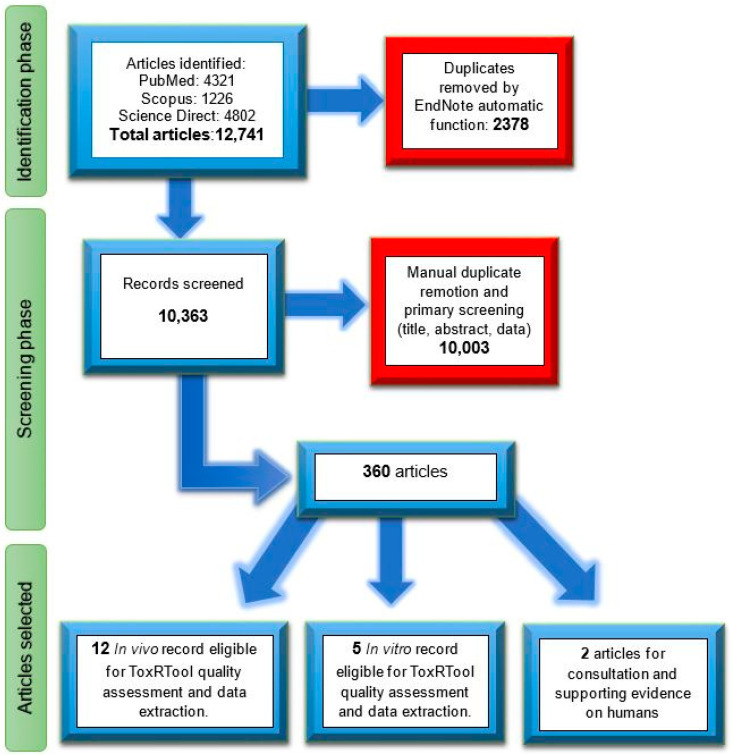
PRISMA graph representing systematic search process for hazard characterization.

The total number of records retrieved from the three databases was N = 12,741. After the exclusion of duplicates and exclusion based on the titles and/or abstracts, 360 records were selected for further evaluations in the second screening. Based on full-text reading, only seventeen articles were considered relevant for the possible derivation of a reference value, as well as one from Kavoosi et al. [65] and one from Chen et al. [66] on human subjects (Figure 2). The quality of the 17 toxicological papers was analyzed by ToxRTool, while the human studies were considered supporting the discussion of the results. No new data were found on TTX analogs, which could help in defining toxicological equivalent factors.

#### 3.1.2. Occurrence in Seafood

For occurrence in edible seafood and in pufferfish, 407 and 93 total records were retrieved, respectively. At the end of the selection process, seven new papers remained on edible seafood and 14 on pufferfish (Figures S1 and S2).

### 3.2. Update of TTX Hazard Characterization

For TTX hazard characterization, data obtained in animal experiments are considered relevant. Indeed, the observed effects are similar to the symptoms described in humans: after acute exposure, skeletal muscle fasciculations, apathy, lethargy, ataxia, ascending progressive paralysis, and death have been observed [67,68,69,70]. Results from in vivo toxicological studies selected after the ELS are described in the text below and summarized in Table 1.

#### 3.2.1. The Re-Assessment of the Abal et al. Study [67]

At the time the EFSA opinion was adopted [16], the available data indicated acute intraperitoneal (i.p.) and subcutaneous (s.c.) LD50 values between 9 and 12.5 µg/kg bw [79,80], while after gavage, much higher LD50 values of 232 µg/kg bw [67] and 532 µg/kg bw [79] were reported. Considering contaminated food consumption as the more plausible and frequent route of human exposure, LD50 values obtained after s.c. and i.p. administration are, by far, less relevant than those obtained after gavage, although a bolus dose (as in the gavage regimen) can still not be fully representative of dietary exposure. Indeed, in the absence of information about kinetics, the fate of TTX in an organism can vary dramatically after different administrations. This was one of the reasons for considering Abal et al. [67] as the key study; in addition, it also provides an acute no observed adverse effect level (NOAEL) of 75 µg/kg bw after a single gavage treatment, based on clinical signs (e.g., apathy) observed at doses ≥125 µg/kg bw. Apathy was indicated as the most sensitive endpoint in this oral acute study in mice. Since, at 250 µg/kg bw, lethality was observed (in four out of seven animals), a relatively steep dose–response curve over a small dose range was suggested, implying quite a high degree of uncertainty and a need to interpret the data with great caution. This was confirmed by the derivation of a BMDL10 = 112 µg/kg bw (where the BMDL is the lower confidence limit of the benchmark dose associated with a 10% response) for lethality as calculated by the EFSA, which was only slightly > the NOAEL for apathy. This was the main reason leading the EFSA to select the NOAEL at the next lower dose tested (25 µg/kg bw). With the application of an uncertainty factor of 100 and using 25 µg/kg bw as the point of departure, an acute reference dose (ARfD) of 0.25 µg/kg bw was derived.

However, the study presented two main limitations. With respect to the indication contained in the OECD Test Guidelines 425 [81] that the authors claimed to follow, the animal observation period was very short (2 h after the treatment) compared to the recommended 14 days (thus loosing possible late effects or recovery), while the fasting period was much longer (with an implication on the absorption of the bolus dose, due to the empty g.i. tract). Therefore, although it was considered a key study, its quality is not optimal, as evidenced by the ToxRTool score (Table 1).

#### 3.2.2. New Acute Toxicity Studies

Since the EFSA opinion, four in vivo acute toxicity studies have been published, unfortunately with a generally moderate quality level, except one, due to flaws in the experimental design, as indicated by the quality score (Table 1). In addition, since the authors used different methodologies, comparison among the obtained results is not easy or even correct.

By using the same protocol and the same animal strain used in the 2017 paper [67], in 2019, ref. [71] focused on macroscopic and ultrastructural alterations in various tissues of several organs (heart, lungs, brain, spleen, liver, kidneys, stomach, and small and large intestines). Necropsy findings indicated that TTX caused stomach swelling 2 h post-administration, despite the absence of further ultrastructural alterations. Transmission electron microscopy (TEM) images revealed an increase in lipid droplets in hepatocytes, swollen mitochondria in spleens, and alterations in the rough endoplasmic reticulum in intestines, distinctive signs of cellular damage. The results suggested the g.i. tract as a possible target for TTX toxicity; however, the limitations described above for the 2017 study also affect this second study since animal sacrifice and necropsy were conducted 2 h after administration. In addition, the discussion about the dose-dependent nature of the effects was quite limited [71].

The differences in TTX toxicity due to various routes of administration (i.p., gavage, and feeding) was studied by Finch et al. [69] in the same strain of mice (SWISS) used by [67,71]; they also tested three different mixtures of STX and TTX, assuming additive toxicity between the two groups of toxins due to the same mechanism of action. Data obtained with the mouse bioassay (i.p. administration), which is no longer used in Europe as an official method, are considered not relevant as they are poorly representative of actual human exposure and are not discussed here. Compared to i.p. administration, the onset of symptoms, death (observed 1–5 h post-dosing), and recovery times were delayed after gavage and feeding. The feeding administration was actually similar to a bolus dose: TTX was administered once, using cream cheese as a vehicle. In addition, no information was given on the possible impact of the cheese, as a highly lipophilic matrix, on TTX stability, bioavailability, and kinetics. The determined LD50 by gavage was ~600 µg/kg bw, while after the ‘feeding’, it was 900 µg/kg bw. The acute NOAEL was determined only after feeding; any change in the behavior, posture, respiration rate, and movement of the mice was observed continuously for 3 h. However, the identified value of 413 µg/kg bw after ‘feeding’ was rather referring to a bolus dose, with the additional uncertainty associated with the ‘unusual’ vehicle.

Wang et al. [70] explored the acute toxic effects of TTX in ICR mice (both sexes) at sub-lethal doses using muscle strength as the major endpoint after oral gavage (250–400 µg/kg bw four doses plus control) and intramuscular injection (4.5–9 µg/kg bw, four doses plus control). Death time and dose-dependent muscle strength variations occurred earlier after the i.m. injection (12–20 min vs. 14–70 min, respectively, after i.m. or gavage). They also estimated LD50 values at 379 µg/kg bw after gavage (250–600 µg/kg bw, five doses plus control) and at 8.6 µg/kg bw after i.m. injection (6–10 µg/kg bw, five doses plus control) (with a steep slope in the dose–mortality curve of TTX). This indicates that the toxicity induced by gavage administration was approximately 44 times lower than that induced by the i.m. injection, confirming that parenteral exposure, such as i.m., s.c., and i.p. exposure [79,80], has a higher toxic potential than oral exposure, likely due to kinetic reasons. The alteration in muscle strength was reversible. Apathy and various degrees of paralysis were reported in all treated animals, independently of the dose; therefore, a NOAEL could not be determined.

Bihong et al. [72] carried out an acute toxicity study in rats with TTX administered in pellets (thus being a real dietary administration); however, since postherpetic neuralgia (PHN) was chemically induced prior to treatment with TTX, the LD50 = 517.43 µg/kg bw should be read with caution. Indeed, control animals without the induction of PHN were not used, making the evaluation of the overall impact of the pathology on TTX intoxication impossible.

#### 3.2.3. New Repeated Toxicity Studies

No subchronic or chronic studies on TTX in animals had been identified at the time of the EFSA opinion [16]. Six in vivo repeated toxicity studies were retrieved by our search, with, unfortunately, all showing limitations in the experimental design and consequently a moderate-to-low quality score (Table 1).

Boente-Juncal et al.’s [68] study reported the effects following 28-day repeated gavage administrations of 25, 75, and 125 µg/kg bw in SWISS female mice. The authors claimed to follow the OECD TG 407 [82], although a number of relevant deviations were noted, among which the use of a single sex (female) and a limited number of animals (unjustified different numbers in treatment groups, with n = 5 as the maximum vs. ten animals per sex requested by the OECD 407) further reduced by death in some groups (with a minimum of two animals in the group treated with 75 µg/kg bw). Therefore, despite the high number of measured parameters, the quantification of results is uncertain. The results are, however, highly suggestive that the repeated exposure of mice to TTX at all the tested doses severely affected the mice’s health status, with an indication of nephrotoxicity and cardiotoxicity [68]; therefore, the NOAEL should have been lower than 25 µg/kg bw. The same authors investigated the effects of TTX at other two doses (20 and 44 µg/kg bw), following the same experimental design with the same limitations, as described above. In addition, the combination of 44 µg/kg bw with different STXs (doses above 5.3 µg/kg bw) were also tested [74]. The results were affected by a high variability, likely due to the low number of animals per group. The authors identified the highest dose as the NOAEL, since according to their evaluation, the ‘*animals did not show any signs of apathy or lethargy during the treatment period*’. However, alterations in blood and urine analysis were reported at both TTX doses; therefore, this conclusion cannot be considered appropriate, and the NOAEL should have been lower than 20 µg TTX/kg bw. The same consideration applies to the administration of the mixture of TTX and STX. The effect of a mixture of TTX and STX after repeated oral administration in mice for 21 days was also studied by Finch et al. [75]. The vehicle used for administering the test item was cream cheese, similarly to the study of acute toxicity from the same group [69], thus presenting the same criticality.

More recently, a subacute study in mice and a subchronic study in rats were also published.

Zhong et al. [76] administered TTX (25, 75, and 125 µg/kg bw) by gavage for seven days to ICR male mice. Two animals’ deaths were recorded at the highest dose, one hour after administration on the third and sixth day. No weights and food intakes were tracked. The authors reported that no other symptoms or behavioral alterations were observed. The results showed an increase in lipid peroxidation and markers of oxidative stress in the liver and the kidneys, at all tested doses, with a dose-dependent behavior. Both the liver and kidneys showed increased inflammation with abnormal levels of tissue damage with specific markers (AST and ALT for liver, BUN and CRE for kidneys). Histological abnormalities were recorded in both organs: namely, appearance of hepatocellular steatosis and the disorganization of liver sinusoid and kidney tubular swelling with the modification of the tissue’s original structure. No NOAEL could be derived since effects were already seen at the lowest dose tested, but the paper provided interesting kinetic data which will be discussed in Section 3.2.4.

Male albino rats were repeatedly exposed for 90 days to 0.5–1 µg TTX/kg bw by an i.p. route; the results were published in two papers reporting the effects on the reproductive system [77] and liver [78]. The animals were not weighted and food intake was not monitored. Furthermore, the i.p. route of administration is quite invasive and can cause distress in animals during long treatment durations; in addition, it is not representative of human exposure and it has been demonstrated by acute studies that TTX toxicity is much higher when the toxin is administered parenterally. Therefore, the results can be interpreted only considering indications for potential hazard and are not suitable for deriving any reference value. Histological damage was also present at the lower dose in both the liver (with a possible induction of lipid peroxidation and the activation of the immune response leading to inflammation and localized necrosis) and male reproductive system (the quality of sperm, tubular necrosis, and hypospermatogenesis).

#### 3.2.4. New Kinetic Information

Since kinetic experiments should be conducted at subtoxic concentrations, and due to the high acute toxicity of TTX, very low toxin concentrations have to be used [66,70,72,73,83]. The compounds can be tracked only for a short period of time before the level drops under the LOD of the analytical method; also, metabolites are difficult to detect [72]. Information has, however, been gathered after TTX administration with various routes of exposure. Some kinetic data were provided by Wang et al. [70]: after the i.m. injection of 7 µg/kg bw in ICR mice, the blood concentration of TTX was 11.56 ng/mL 5 min after toxin administration, and then it gradually declined until after one hour, when TTX could barely be detected. This suggests that a peak was reached in a very short time. As expected, after gavage (300 µg/kg bw), the TTX concentration increased during the first 45 min, reaching a peak (Cmax = 9.41 ng/mL), and then declined to 4.44 ng/mL after two hours. Differences were likely due to the delayed absorption and elimination of TTX after oral administration.

After a single gavage of 75 μg/kg bw with the same strain of mice (ICR), samples (blood, liver, kidney, and small intestine) were taken after 2 h, 4 h, 6 h, 12 h, 24 h, 48 h, 72 h, and 168 h [76]. The Cmax in the blood was reached 2 h after ingestion and then the concentration decreased over time, indicating that, in mice, TTX is quickly distributed to the blood after ingestion. This was confirmed by the Cmax in the liver (72.67 ng/g), reached 2 h after ingestion. Since TTX was still present after 168 h (2.30 ng/g), despite a time-dependent decrease in TTX concentrations, the elimination kinetic was not as rapid as the uptake. A similar trend was observed in the kidney and small intestine. Only the parental compound was reported.

The distribution of TTX in the liver, kidneys, blood, small intestine, and feces was also measured after 7 days of gavage administration at three TTX dose levels (25, 75, and 125 μg/kg bw). The TTX concentration in mouse tissues was not proportional to the administered dose, suggesting that there could be saturation in the absorption; indeed, the highest levels were measured in the small intestine, the second highest in the liver, the third highest in the blood, and the lowest in the kidneys. To corroborate this hypothesis, the TTX concentration in the feces was much higher than that in other tissues, likely associated with non-absorbed TTX. The fecal content of TTX could not be distinguished between the fraction which could have potentially been eliminated by bile and the fraction reaching the feces due to a lack of absorption. As TTX is already a very hydrophilic substance, it is more plausible that the main excretion route is with urine, as indicated by other studies in humans and animals [66,73] and in vitro [83].

Bihong et al. [73] found interesting results using labeled 11-[3H]-TTX administered as a single i.m. dose (6 µg/kg bw) in SD male and female rats. No sex difference was reported for any kinetic parameters. The Cmax in the plasma was reached within 10 min afterwards and radioactivity was below the detection limit in the plasma after 24 h, with a half-life of 2.31 h. The average total radioactivity in the stomach, lungs, kidneys, and intestines was higher than the plasma concentration after 48 h. Bile secretion accounted for 0.43%, and approximately 51% of the dose was recovered in the urine, the predominant route of elimination of the parent compound; this was as expected, considering the high hydrophilicity of TTX. Oxidized TTX was the only identified urinary metabolite. Unfortunately, the mass balance indicated a loss of about 30% of the administered radioactivity (only 69% recovery), casting doubts on the quantification of many parameters. This was likely due to hydrogen–tritium exchange in the rats with the production of tritiated water, excreted in the breath and saliva. This, together with the parenteral route of administration, limits the relevance of the study.

Information on TTX excretion was obtained in vitro in a porcine renal proximal tubule epithelial cell line (LLC-PK1), using 50 µM TTX (which is quite a high concentration) [83]. From the model, TTX transport seemed to be both a transcellular and carrier-mediated process; the amount of TTX excreted was approximately 20% of the administered value at 37 °C, but it is not possible to conclude on the possible saturation of the involved transporter(s) since a single high dose was tested. By using specific inhibitors of the various carriers, TTX resulted in being primarily excreted by organic cation transporters (OCTs) and organic cation/carnitine transporters (OCTNs), partially transported by organic anion transporters (OATs) and multidrug resistance-associated proteins (MRPs), and negligibly transported by multidrug and toxic compound extrusion transporters (MATEs).

When 10 µg of TTX was administered intravenously to eight healthy human volunteers, TTX was determined in human urine with an HILIC MS/MS technique [66]. Human samples were gathered every 4 h for the first 12 h and then after 24 and 36 h. From the results, the cumulative excretion of TTX in the first 12 h was 38.34 ± 5.19% of the initial dose. The excretion dropped in the next 12 h as the toxin excreted between 12 and 24 h was registered as only 3.67 ± 2.56%. No information on the urinary levels between 24 and 36 h was available. The about 57% of undetected toxin could have been due to different reasons not discussed by the authors; no metabolites were investigated and the performance of the method could have been limited (the recovery was not reported or a second phase of very slow elimination was present). The study also suggested that TTX excretion occurs mainly via urine in humans without providing any new additional data. Besides possible ethical concerns, no indications were given about the effects of TTX on human volunteers, even if 10 µg injected in a vein (and therefore 100% bioavailable) could be relevant considering the ARfD of 0.25 μg/kg bw derived by the EFSA [16]. This is equivalent to an intake of 17.5 µg of TTX for an adult of 70 Kg. Considering that the absorption and the bioavailability after oral exposure is lower, the presence (or the absence) of any effects can lead to gathering important information.

Based on the available data obtained in vitro on neuronal cells, as well as in vivo data on rats and mice upon oral and parenteral dosing, a PBK model was developed, making use of in vitro/in silico quantitative in vitro/in vivo extrapolation to evaluate the predictions. The results indicated that the predicted dose–response data matched the data from rats and mice in in vivo studies. Therefore, the PBK modeling-based reverse dosimetry of data on in vitro TTX effects can adequately predict the in vivo neurotoxicity of TTX in rodents, providing a novel proof of principle for this methodology [84].

#### 3.2.5. In Vitro Studies Supporting the MoA

Some interesting in vitro studies not included in the EFSA review were considered as possible supporting information on TTX toxicity and therefore also evaluated with the ToxRTool (Table 2). Of these, the most interesting are discussed in the following section. They are mainly devoted to elucidating the mechanism of action.

As an example, the binding of TTX and its derivatives to voltage-sensitive sodium channel subtypes (Na_v_ 1.1 to Na_v_ 1.7) was analyzed [86]. The inhibitory concentrations of TTX on Na_v_ 1.1– Na_v_ 1.7 were extremely variable (Na_v_ 1.1: 4.1 nM; Na_v_ 1.2: 14 nM; Na_v_ 1.3: 5.3 nM; Na_v_ 1.4: 7.6 nM; Na_v_ 1.5: 1000 nM; Na_v_ 1.6: 2.3 nM; Na_v_ 1.7: 36 nM), in accordance with those previously reported by Rosker et al. [51]. Interestingly, some TTX analogs, i.e., the 5-deoxy-10,7-lactone-type analogs and 4,9-anhydro-type analogs, did not cause any inhibition. However, possible differences in the kinetics of the derivatives might limit their relevance in using their binding potency for TEF determination.

The use of micro-electrode array (MEA) recordings as an integrated measure of neurotransmission allowed researchers to demonstrate that TTX inhibited neuronal electrical activity in both primary rat cortical cultures and human-induced pluripotent stem cell (hIPSC)-derived iCell1 neurons in a co-culture with hIPSC-derived iCell1 astrocytes [85]. The IC50 values of 7 and 10 nM, respectively, suggested that interspecies differences were limited for TTX. The dose–response curve was quite steep. However, it is necessary to consider that TTX was directly administered to neurons, without the protection of the BBB.

Human cerebral organoids were exposed for 24 h to 0.1, 1, and 10 nM TTX; transcriptomic alterations were reported [88]. The brain mainly expresses Na_v_ types that are highly sensitive to TTX (IC in nM range); effects were recorded only at 10 nM dose with a decrease in cell viability and alterations in several types of RNAs associated with the modification of cation channel expression and synapses homeostasis. However, the cerebral organoid was developed omitting the presence of a BBB, and the direct application of TTX to the brain doe not consider possible kinetic contribution and offers few advantages with respect to the 2D cell model described above.

#### 3.2.6. Available Human Intoxication Data 

TTX is undergoing clinical evaluation as a highly specific voltage-gated sodium channel (VGSC) blocker, with a potential application as a peripheral-acting analgesic for chronic pain [65]. For this process, information on the cardiac liability of TTX at therapeutic-relevant concentrations in twenty-five healthy adults was collected. The randomized, double-blind, and placebo- and positive-controlled study assessed single subcutaneous doses of 15 µg, 30 µg, and 45 µg of TTX over three periods, with a 7-day washout between each period. The Cmax was achieved within 1.5 h proportionally to the dose. Concentration–QTc analysis revealed no alteration in the ECG in both the frequency and morphology of Q and T waves, meeting the criteria of a negative QT study. Safety assessment showed an overall occurrence of less than 5% in the TTX treatment groups of paresthesia, oral paresthesia, headache, dizziness, nausea, and myalgia, but no other clinically relevant changes were reported.

One of the major problems in TTX risk assessment, as also underlined in the EFSA opinion [16], is the lack of solid human intoxication data. Guardone et al. [7] examined papers listing intoxicated people, their geographical location, and the source of intoxication (at least the category of seafood) caused by TTXs. In 3032 registered cases all over the world (with the exclusion of Japan), 2641 (87.1%) were diagnosed by anamnesis and only 375 relied on a proper analytic detection, reporting the used method. In 16 cases, TTX presence was confirmed without specifying the analytical method used. As TTX can coexist with STXs, the analytical detection of TTX with a reliable method like HPLC/MS is necessary to attribute the intoxication to the toxin, due to the symptoms being identical. The mouse bioassay cannot be considered valid anymore, due to both its low sensitivity and ethical concern and because it does not discern between different toxins. In only 1335 (44%) cases, sex was reported, and males (898 cases) were more frequently involved than females (437). Age was reported only in 304 (10%) cases, covering a large age interval from 4 to 74 years. Lethality involved 341 cases, with significant differences between fish products (16.3%) and other product categories like gastropods (4.9%), arthropods (2.4%), and cephalopods (2.2%). No deadly intoxications by the ingestion of edible bivalves were recorded. The highest number of cases was recorded in Asia, with 2686 cases and a lethality around 10.4%, but in other geographical locations (including Europe and North America), the fatality rate was higher: it was 21.4% in Europe (3/14 cases) and 30.4% in North America (7/23 cases). Fishes were the main sources responsible for human intoxications, with 1817 cases (59.9%), followed by gastropods (634 (20.9%)), crabs (492 (16.2%)), and octopuses (89 (2.9%)), while bivalves were not responsible for any TTX intoxication. Most recent cases confirm that pufferfishes are the most common cause of TTX poisoning [89,90,91].

However, despite the high number of cases analyzed, human data cannot be considered suitable for the derivation of reference values for TTX due to many gaps in the records (especially on sex, age, and the absence of analytical tests on both patients and leftover food, when available).

#### 3.2.7. Conclusions on New Toxicity Studies

Although a number of in vivo studies have been published since the adoption of the EFSA opinion, their limited-to-moderate quality hampers the possibility of indicating any of them as a key study for the identification of acute toxicity. Although some of the authors claimed to follow the OECD TG 425 [81] for acute toxicity, a number of relevant deviations (e.g., the regimen, the fasting period, the number of animals, or the vehicle for administration) were included, which could greatly impact the final outcome.

Overall, based on LD50 values, TTX toxicity is much higher following parenteral administration when compared to the oral route, thus highlighting that kinetics may largely affect TTX toxicity. It is evident that after i.m. injection, TTX detection in blood is very rapid, with a Tmax (5–10 min vs. 45 min–2 h) attained in a shorter time interval when compared to that from gavage [70,73,76], and the half-life is consistently lower. After gavage, the blood concentration is not proportional to the administered dose, suggesting that there could be saturation in the absorption, supported by distribution in the organs with the highest level measured in the small intestine (and hence in the feces), the second highest in the liver, the third highest in the blood, and the lowest in the kidneys. The presence in the feces is likely attributable to unabsorbed parent compound, since TTX is mainly excreted in urine [66,73]. The involvement of active transport with a renal proximal tubule epithelial cell line [83] suggests that g.i. TTX absorption can be mediated by saturable transporters, explaining the lower bioavailability obtained after its oral administration. Unfortunately, no information is available on TTX biotransformation.

Results associated with oral administration are therefore more relevant for the derivation of any reference value; gavage, being a bolus dose, is less representative of human exposure when compared to dietary or feeding studies. However, no good dietary or feeding studies are available, since the two studies that claimed to use a feeding regimen administered TTX dissolved in cream cheese, which was actually a bolus dose. Furthermore, the cream cheese should have had some impact on bioavailability/absorption in the intestine, since the comparison between gavage and ‘feeding’ showed a ~50% higher LD50 value (900 µg TTX/kg bw vs. 600 µg TTX/kg bw) for the latter. Therefore, among the available studies, the ones using gavage are considered the most relevant ones.

After gavage, LD50 values in mice have ranged from the lowest value of 232 µg/kg bw reported by Abal et al. [67] (despite the short observation time) to the highest value of 600 µg/kg bw derived by Finch et al. [69], passing through intermediate values such as 379 µg/kg bw [70] and 532 µg/kg bw [79]. This large variability can be attributed to the different methodologies used and the mouse strains. Regarding the identification of an acute NOAEL, the only additional value (413 µg/kg bw) was obtained by Finch et al. [69], but due to the limitations of the study, it should be taken with caution.

Despite reasonable expectations to define a TTX limit value that protects human health from risky exposures and at the same time does not penalize the important seafood industry [22], this thorough toxicological review of the new studies published since the EFSA opinion does not allow the limit expressed by the EFSA to be exceeded. Considering the uncertainties and the steepness of the dose–response curve, applying a conservative approach, the lowest LD50, and the acute NOAEL values from Abal et al.’s [67] study as point of departure still seems to be the best choice, since new published data are not robust enough to justify any modification.

Regarding repeated toxicity, the subacute NOAEL after gavage in mice seems to be lower than about 20 µg TTX/kg bw per day, as indicated by a seven-day study [76] and two studies with TTX administered for 28 days [68,74]. Indeed, in all studies, effects were also reported at the lowest dose tested, indicating the induction of nephrotoxicity, cardiotoxicity, and hepatotoxicity. Although the quality of the studies was moderate, the obtained results seem to be consistent. Data on rats were obtained after 90 days of treatment via the i.p. injection of 0.5–1 µg TTX/kg bw, and effects were observed at all doses. However, the results are not suitable for deriving any reference value as the administration route is poorly relevant [77,78].

### 3.3. Occurrence of TTX in Seafood in Europe

In 2017, the EFSA pointed out that a low number of studies considered TTX occurrence in European bivalves and gastropods. The main areas screened for TTXs were England, Portugal, and Greece, and some data were from the Netherlands and United Kingdom (see [16]). The upper-bond 95th percentile concerning all received data on TTX in bivalves was 28 μg/kg and the highest reported level among all data was 253 μg/kg in oysters.

**Figure 3:**
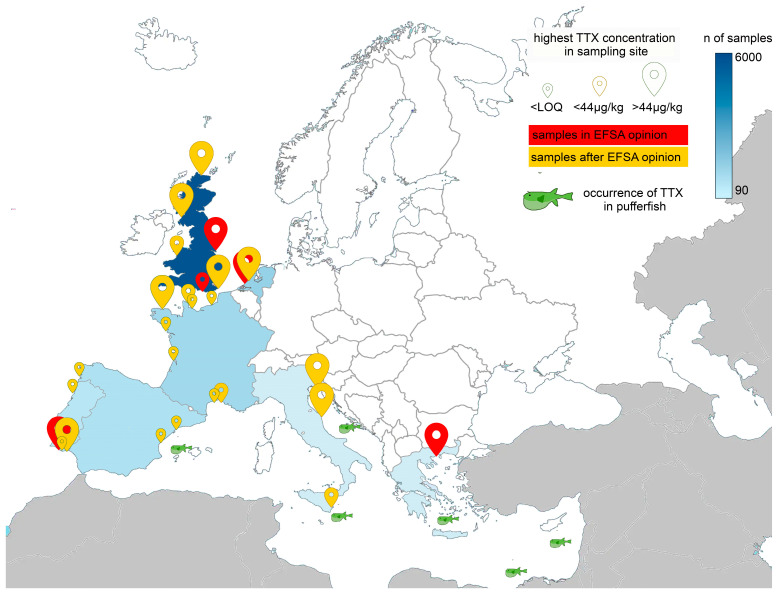
TTX occurrence in European seafood and pufferfish.

After the publication of the EFSA opinion, several new studies were reported (Figure 3), and in 2021, the occurrence data were updated by an ELS, showing increasing attention to the presence of TTXs in live bivalve mollusks captured in European sea waters [22]. The review showed that data were still fragmented and available only for few European countries (the UK, France, The Netherlands, Italy, and Spain). 

The highest TTX concentration was registered in *Mytilus galloprovincialis*, with concentrations up to 541 μg/kg in Italy, and in *Crassostrea gigas*, with concentrations up to 331 μg/kg in Italy and 253 μg/kg in the Netherlands and the UK [22] (Table 3), values well above the 44 μg/kg suggested by the EFSA as a safe level.

At the time of the review, no positive samples had been found in Spain [29,33], and the authors had already pointed out the need for broader and more frequent sampling; however, no other data have been published since then. The Netherlands, which has formally included TTX in its shellfish monitoring program and used the value of 44 μg/kg suggested by the EFSA as an action limit since 2022 (https://www.wur.nl/en/research-results/research-institutes/food-safety-research/reference-laboratory/national-reference-laboratory/marine-biotoxins.htm (last accessed on 20 December 2024)), started a national control campaign for TTX in 2015 and by the time of the review [22] had already analyzed 1063 samples from all shellfish production areas, implementing a representative weekly sampling program but only between June and October, 2015–2017. They found the highest concentrations of the few (31) positive samples in June 2016, with 253 μg TTX/kg in oysters and 101 μg TTX/kg in mussels and no TTX analogs [20]. To our knowledge, no new data are publicly available.

In France, the data reported by Antonelli et al. [22], from the analysis of few samples in spring/summer, showed a safe situation, with positive samples spotted, always below the EFSA limit. To have a better coverage of the natural toxins in mollusks, the program EMERGTOX was set up along the French coasts in 2018 to quantify regulated and unregulated lipophilic and hydrophilic toxins. In a five-year period, TTXs were not detected in 436 samples (personal communication) analyzed in the EMERGTOX framework [95]. However, the authors believe that this may have been due to the (i) high LOD (11 μg/kg for TTX) of the multi-toxin method used, (ii) lack of a purification step, and (iii) likely low concentrations observed so far in France (necessarily below the LOD). A significantly higher LOD does not allow us to compare French data when the frequency or the percentage of positive samples out of the total samples analyzed is concerned.

Significantly higher concentrations were found in France in 2021 in Pacific oysters, *Crassostrea gigas*, deployed in cages, from April to September in an estuary where TTX was previously detected (the sampling point was not included in the EMERGTOX program) [28]. Positive samples were reported from late June to July, with concentrations decreasing over time in the digestive gland from 424 to 101 μg/kg (equivalent to 74 and 17 μg/kg of total flesh). This observation confirms the tendency, reported in several studies, for TTXs to be detected during late May, June, and July before disappearing but also that it is still necessary to extend the geographical coverage of sampling to discover areas or habitats where TTX can accumulate in mollusks, until more data on the mechanisms of TTX production are available. Sporadic samples with concentrations over the LOD were also collected during winter (up to 20 μg/kg) [30,36]. This suggests that this season should be included in a sampling program, because it cannot be excluded that, in some cases, the toxin could be high even in winter, especially from the perspective of sea-surface warming after climate change.

Even if seasonality is frequently observed, little information can be derived when considering associations with environmental parameters (temperature, salinity, depth, pH, nutrients, etc.). In the UK, to better understand the temporal distribution of the toxin, four shellfish sites were monitored monthly over a five-and-a-half-year period (202 μg/kg was the highest level of TTX recorded in July) [30]. The accumulation of TTX in bivalves was seasonal every year, with higher concentrations between June and August, generally reaching a peak by the end of June or beginning of July. This period coincided with the sea-surface temperature increase after winter (up to 15 °C), but it is to be noted that further increases in temperature did not lead to further increases in the TTX concentration. On the contrary, by the time the temperature reached its peak in July and August, TTX concentrations had decreased, suggesting that, for TTX accumulation, there could be an optimal temperature range between 15 and 20 °C in production areas with shallow waters where larger and more rapid shifts in temperature can occur. However, temperature is unlikely to be the only factor affecting TTX accumulation [30].

To better investigate these aspects and increase knowledge on TTX occurrence in Italy, the Marano Lagoon (Venice, Italy) was identified as a hotspot for conducting field surveys on the topic with weekly sampling from May to July and then monthly sampling until September [25]. The researchers found a positive association between seawater pH and temperature and TTX occurrence in mussels and oysters, with the highest amount detected in June, in mussels, with a value of 276 μg/kg. No TTX analogueswere detected in oysters, while in mussels, the most relevant were 11-deoxy-TTX/5-deoxy-TTX and 6,11-dideoxy- TTX, which reached their highest levels in June (421.9 and 346 µg/kg, respectively). The authors confirmed seasonality in the TTX accumulation in the mollusks and suggested that environmental factors likely play a role in the accumulation of TTX in the bivalves, possibly also through some influence on the bacterial communities of the TTX-positive bivalves analyzed [25].

The Conero Riviera, in the central Adriatic Sea, has witnessed similar outcomes since mussels were found to have significant TTX contamination levels in the spring/summer period (9–296 µg/kg), with concentration peaks in June and very variable levels of TTX from one sample to another [27]. The presence of the toxin in other organisms as well (flatworms, bacteria, and mesozooplankton) and its distribution in mussel tissues strengthen the hypothesis of the exogenous origin of TTX [27]. From these two studies, the number of positive samples in Italy seems quite high (30% in Antonelli et al. [25] and 25% in Bacchiocchi et al. [27]) with respect to other countries. However, it should be considered that, on one hand, samples were collected only in the time frame from April to September, the period in which TTX occurrence was detected, and the number of samples was lower than in other studies. A higher number of sampling sites should be studied over the year, not limited to the warm season, to have more reliable data.

For gastropods, very few data come from Portugal, where very high levels of non-edible viscera (42.1 mg/kg of TTX and 56.3 mg/kg of 4,9-anhydroTTX) in the trumpet shell, *Charonia lampas*, were found. In the edible part, much lower levels were detected (31.3 and 88.0 μg/kg of TTX and 4,9-anhydroTTX), still higher than the EFSA limit [97]. Despite the high levels found, few data are available on TTX in bivalve mollusks in Portugal. Twenty samples, collected between April and September, 2015, from classified shellfish-producing areas, were found to be TTX-free [96]. Subsequent data from 2018, from 117 samples of bivalves collected weekly from May to October in classified production areas on the south and southwest Portuguese coast, confirmed low or no concentrations of TTX. Neither TTX nor any of its analogs were detected at quantifiable levels (LOQ: 16 μg TTX/kg), while TTX trace levels were observed in only three mussel samples collected in July and August [35]. However, a picture of the whole year is missing, and it is plausible that, using a more sensitive method, the percentage of positive samples would be higher, even if at levels below the EFSA’s recommended value. 

Finally, it is also worth noting the growing presence of fish from the Tetraodontidae family in the Mediterranean area (Table 4). Although fishing and marketing them is prohibited, illegal or uninformed consumption cannot be excluded; some isolated cases of fishers’ intoxication in North Africa have been reported, associated with the many specimens of pufferfish settled in the Mediterranean Sea [89,90].

Dating back to 2012, the FAO reported an increasing presence of the species *Lagocephalus sceleratus* in the eastern Mediterranean area, following its entry through the Suez Canal [113]. This fish can adapt easily to new habitats, and now it is regarded to be among the worst invasive species in the Mediterranean Sea. Indeed, the number of sightings is experiencing a significant increase in the whole Mediterranean Sea: in Greece [102,114], Turkey [110,115], Tunisia [116], Lebanon [113], Malta [117], Italy [118,119], Spain [99], and Croatia [120]. Several relevant toxic Lagocephalus species (*L. sceleratus, L. suezensis,* and *L. torquigener flavimaculosus*) have been described. In the area of the Aegean Sea, in Turkey, Cyprus, and Greece, the presence of *L. sceleratus* is the most frequent, with TTXs levels up to 86 mg/Kg in muscles and up to 535.78 mg/kg in gonads, considering the sum of TTX and its analogs [104,105,111].

In the rest of the Mediterranean, pufferfish specimens tested seem to have lower contamination levels. Specimens from the Spanish cost revealed high concentrations of TTX equivalents (up to 1 and 26 mg/Kg in muscles and gonads, respectively) in *L. sceleratus*, while no TTXs were identified in *L. lagocephalus* and *Sphoeroides pachygaster* individuals [99]. Instead, in another study, the 20 fish analyzed, of the species *S. pachygaster*, of the 56 caught along the Italian coasts (Straits of Sicily) were negative for TTXs in all tissues tested [100]. 

### 3.4. Food Consumption Data

Consumption data from the EFSA Comprehensive European Food Consumption Database were extracted, after selecting appropriate filters. For countries, no filters were applied. All available surveys were used, and all population groups, divided into adolescents, adults, elderly people, infants, other children, toddlers, and very elderly people, were included. To narrow the research and select categories useful for our purpose, the parameters selected from exposure hierarchy L1 to exposure hierarchy L7 (from a pull-down menu) were the following:

Exposure hierarchy L1: (i) Fish, seafood, amphibians, reptiles, and invertebrates.
Exposure hierarchy L2: (i) Mollusks, (ii) Fish, and seafoodprocessed.
Exposure hierarchy L3: (i) Clams, cockles, arkshells, (ii) Mussels, (iii) Oysters.
Exposure hierarchy L4: All.
Exposure hierarchy L5: (i) Blue mussels, (ii) Clams, (iii) Oysters, (iv) Scallops, pectens.
Levels 6 and 7 were equal to level 5.

Only data with statistical significance were used. Only five countries had significant 95th-percentile consumption values (Table S2): France, Italy, Spain, Portugal, and Sweden. All of them but Sweden, which had data for adolescents, had data for adults. The highest consumption was for Italian adults, consuming portions of 161.98 g of mussels, followed by the French and Italian consumption of ~135 g per portion of oysters and mollusks, respectively. This is in line with the substantial tradition of consuming shellfish delicacies in the two countries, with a specific local survey indicating that consumption is around 150 g of shellfish in northeast Italy [121].

### 3.5. Risk Assessment

The current proposed group ARfD (acute reference dose) for TTXeq (including TTX and its analogs) is 0.25 µg/kg bw. Based on our re-evaluation of the studies published after the adoption of the EFSA opinion or not considered by the EFSA due to the time constraints of their literature search, it appears that new data are not robust enough to justify any change. Therefore, considering the uncertainties and the steepness of the dose–response curve, the ARfD derived by the EFSA still seems to be the best choice.

To assess cases of large-portion consumption, the EFSA 2017 CONTAM panel assumed a single meal of shellfish to be 400 g, as used in previous opinions on marine toxins [18]. A concentration below 44 µg of TTXeq/kg of shellfish meat, based on such a large portion, was considered by the EFSA the maximum concentration allowed in bivalves not to result in adverse effects in heavy consumers.

As discussed in Section 3.3, data on TTX occurrence in bivalves (mussels and oysters) in Europe from 2014 to 2022 are still difficult to compare and in many cases not representative of the countries (few stations for sampling; only seasonal sampling; different LODs and LOQs; etc.). However, when available, the highest data, usually from the spring/summer season, are much higher than the EFSA limit of 44 µg TTXeq/kg. In addition, the few data available on gastropods show that the edible part of the organism can accumulate a very high concentration of TTXs and, indeed, most of the samples exceed the EFSA limit. Therefore, significant concern for heavy consumers can be expressed, even if it seems limited to the warmer months, that is, for a short period in the year.

It can be inferred that a portion of 400 g is poorly representative of the general population, since for countries where data were available (Table S2), the consumption ranged from 18.67 g (adolescents in Sweden) to 161.98 g (Italian adults). Therefore, to perform a risk assessment on the general European population (not heavy consumers, 95th percentile), we consider the highest food portion of 162 g of mussel flesh and a mean adult body weight of 70 kg.

Based on this, the ARfD per person is 0.25 µg/kg bw × 70 kg = 17.5 µg; the same person is assumed to consume 162 g of mollusk meat.

The calculation of the total TTX intake to be then compared with the ARfD per person is performed with the following contamination levels, for bivalves:

1. The highest value of contamination measured in bivalves (541 µg/kg, worst-case approach) gives rise to an intake of 541 µg/kg × 0.162 kg = 87.6 µg, largely exceeding the ARfD per person. To visualize the level of concern, we also calculate the margin of safety (MoS), defined as the ratio of the no-observed-adverse-effect level (NOAEL) obtained from animal toxicology studies (25 µg/kg bw) to the estimated human exposure level. In this analysis, the MoS is approximately 20, which is significantly lower than the minimal acceptable MoS of 100, hence confirming a potentially significant concern for consumer safety.
2. The average highest value of contamination measured in bivalves reported in each study (no study gave the average value of µg TTX/kg) (~104.4 µg/kg) gives rise to an intake of 104.4 µg/kg × 0.162 kg = 16.9 µg, similar to the ARfD per person. Accordingly, the calculated MoS is 103, indicating that this level of contamination represents a borderline case, to be looked at with caution considering the uncertainties in the ARfD derivation.
3. The median highest value of contamination measured in bivalves reported in each study (~16 µg/kg) gives rise to an intake of 24 µg/kg × 0.162 kg = 3.9 µg, well below the ArfD. The MoS in this case is 448, confirming no concern for consumers.

The worst case for the ingestion of gastropods (*Charonia lampas*) for a regular consumer would be 966.3 µg/kg × 0.162 kg = 157 µg, almost double the value of the worst case for bivalves, much higher than the ARfD per person and showing a MoS close to 10. Indeed, the only known case of intoxication in Europe was in Portugal after the ingestion of *Charonia lampas*.

Therefore, even for the general population, a significant number of samples analyzed during the warmer season are characterized by levels of contamination that could be of concern for consumers. Considering that TTX analogs’ contribution is not known at the moment, the potential risk could be even higher.

A reliable risk assessment for a single country can be presently performed only for the UK, where the data cover a significant number of sites during a quite long time span (2014–2018). The worst case is 253 µg/kg × 0.162 kg = 40.9 µg, about double the EFSA limit. This improves when considering the average TTX concentration in positive bivalves (153 µg/kg × 0.162 kg = 24.8 µg) but is still higher than the limit. Since positive samples are only a very small percentage of the total samples, with high values occurring between July and August, to protect the population from risky exposure and to monitor possible variation in TTX occurrence, monthly monitoring during winter and more frequent monitoring in the period of higher exposure in the known areas of TTX occurrence is reliable. For other countries, the assessment performed on the general European population can be applied, at least until more systematic data on occurrence, covering the whole year, are available.

Finally, the extremely high values of TTX found in the muscles of pufferfish (mg/kg), now established as permanent inhabitants of Mediterranean Sea, raises serious concerns. With the potential of uninformed consumption or the growing population in Europe from Asian backgrounds, who may have different dietary habits compared to Caucasian European people, the consideration of pufferfish as a potential source of exposure is critical. Indeed, assuming a portion of fish of 200 g (the size of a reared fish), in the worst-case scenario observed in Cyprus, the ingestion of TTX would be 8340 µg TTX/kg × 0.200 kg = 1668 µg TTX, with a MoS = 1. To mitigate this risk, extensive communication campaigns are essential to educate the public about the dangers associated with consuming these exotic fish. Awareness efforts should emphasize the potential health hazards, ensuring that individuals understand the risks and can make informed dietary choices.

## 4. Key Insights and Outstanding Challenges

TTX in temperate areas should increase in upcoming years, due to reasons including climate change, but it is not among the marine biotoxins for which there are reference limits, so it is crucial to improve the awareness and preparedness of different stakeholders through the dissemination of information and knowledge on TTX exposure and possible associated risk.

This analysis of new studies published after the EFSA opinion [16] demonstrates that it is not possible to decrease the uncertainties in the ARfD derivation yet and that good-quality data to describe the kinetics and the toxicity of TTX are still a very important gap. It seems necessary to underline the use of oral dietary administration, the relevant exposure route for humans, possibly using harmonized and internationally accepted methodologies, such as the OECD TG, without introducing arbitrary deviations impacting the final outcome. This cost-effective approach will allow the use of results for risk assessment, without wasting animals, time, money, and manpower. The possibility of relying on in silico information, PBK modeling, and QIVIVE is very promising and could, in the future, decrease the use of animals in in vivo studies.Clarifying kinetic behavior could help in extrapolating data on exposure from route to route, as well as from low to high doses and from animal or in vitro data to human data.

On the other hand, TTX occurrence data in seafood are still geographically (few sampling sites) and temporally (mostly summer months) limited and poorly comparable for different LODs/LOQs. As a consequence, it can be recommended that whenever occurrence data are reported for seafood, it is extremely important to detail the sampling plan, specifying the time and site of sampling, considering more representative coastal areas (e.g., where fish or bivalve farms are located), and possibly spanning over the year (in some areas, samples also show positive results during cold months) and measuring some environmental parameters such as temperature, salinity, or pH. In addition to the number of samples, the corresponding concentrations of TTX should be reported, not simply giving the range or the average values; this will allow us to understand the seasonality (which can be different depending on the latitude) and the possible association with temperature or other parameters. It is also important to use validated analytical methods with an appropriate LOD/LOQ, to allow comparisons among different studies also in terms of the % of positive samples.

In addition, the acquisition of information on the ecological mechanisms underlying the selection and bioaccumulation of bacterial/phytoplanktonic species producing TTX and the taxonomic identification of the producing species will pave the way for the development of early warning systems. The observed correlation between sea temperature, pH, and TTX accumulation in bivalve mollusks underscores the need for future surveillance efforts to incorporate these environmental variables, with the aim of understanding the triggers promoting TTX presence. Furthermore, understanding depuration kinetics in seafood would support the development of predictive models to anticipate high risk and enable the implementation of appropriate mitigation measures such as temporary fishing bans or recovery periods to allow for the complete depuration of seafood prior to commercialization.

According to our analysis, a significant number of bivalve samples analyzed during the warmer season are characterized by levels of contamination that exceed the EFSA-derived ARfD per person. In addition, the available data indicate that ingestion of gastropods should also be considered, also taking into account that the levels of contamination found seem to attain much higher values. Starting from this observation, it can be commented that although the situation seems to be of high concern based on the still-limited database on occurrence, no intoxication events following bivalves’ consumption have been reported so far in Europe. There are a couple of reasons for this: first of all, the ARfD suggested by the EFSA was derived with a very conservative approach, due to the high uncertainty of the data available; in addition, there is limited awareness about the likelihood of this kind of intoxication, even amongmedical personnel, and symptoms can be easily attributed to other causes, since analysis of potentially contaminated food residues is not generally carried out. On this latter point, physicians, pharmacists, and local health authorities should be informed on the issue and trained in recognizing symptoms and defining cases to track intoxication events; diagnosis should be paralleled by confirmation with analysis of food items with appropriate analytical methods also evaluating the co-presence of other toxins like STX, which shares symptoms with TTX. Registration forms for potential cases should be available, detailing information such as age, sex, food consumed, and the investigation of TTX concentration in food leftovers, if available, to assess toxin concentration.

As outlined in the Italian guidelines for the management of *Ostreopsis ovata* presence [122], monitoring alone may be not enough to prevent the general population from dangerous exposure. It is not feasible to plan frequent sampling and it is not possible to avoid the consumption of any potentially contaminated seafood. For this reason, it is pivotal that all stakeholders (seafood producers, fishery associations, health personnel and local authorities, and citizens) are informed about the possible risk associated with the presence of TTX, including at our latitudes, to make them aware (a condition which can influence their actions and the rapidity that could be taken individually or collectively to address exposure and vulnerability to hazards) and prepared in order to take direct and indirect measures to reduce damages that accompany events to the minimum possible level. This will also allow us to limit the possible illegal or uninformed consumption of pufferfish, which is uncommon so far but to be considered with respect to citizens of Asian origin, who have different dietary habits and traditions.

The detection and description of the toxicity potential of TTX analogs are other issues that should receive more attention in the future. At the time of the EFSA opinion [16], the panel noted that the derivation of TTX analogs’ relative potencies was associated with high uncertainties, and the situation is currently not changed since few papers have dealt with this issue. Although the data available so far suggest that TTX is the most acutely toxic among the known variants, there is no chance to score among analogs or to attempt the derivation of any Toxicity Equivalent Factor (TEF). Therefore, the ARfD of 0.25 µg/kg bw has to be considered a group ARfD whenever the sum of TTX and its analogs are detected.

In addition, the co-occurrence in the environment and in seafood for human consumption of TTX and any other chemical (human-made or natural) sharing the same targets (e.g., blocking voltage-gated sodium channels) should be carefully investigated, since their toxicity could be additive.

## 5. Concluding Remarks

This review of recent data highlights the complexity of TTX toxicity and its occurrence in seafood, underlining the importance of continued monitoring and research. Despite advances in our understanding, critical knowledge gaps persist in the areas of toxicity and toxicokinetics, where the quality of produced data is crucial. Leveraging innovative approaches such as physiologically based kinetic modeling (PBK) and in vitro and in silico tools offers the potential to reduce reliance on animal testing.

Adequate human exposure data are also lacking; robust and harmonized methodologies, as well as standardized sampling protocols and analytical techniques, are essential to generate reliable data for risk assessment, especially in underrepresented areas.

The role of environmental factors, such as climate change, in the increasing prevalence of TTX in European waters cannot be underestimated, with the aim of predicting, by fit-for-purpose modeling, TTX presence to implement appropriate management strategies and reduce the risk of commercializing TTX-contaminated live bivalve mollusks or any other seafood.

Public awareness campaigns are crucial to mitigate this risk and to inform stakeholders and consumers, especially regarding the dangers associated with the consumption of highly contaminated pufferfish as their populations continue to expand in the Mediterranean.

Ultimately, balancing consumer safety with the sustainability of the seafood industry requires a coordinated approach across European regulatory bodies. Harmonized legislation, informed by the latest scientific evidence, is paramount to addressing the challenges posed by TTX contamination.

## Figures and Tables

**Table 1:** Relevant toxicological data extracted from the articles on TTX toxicity from in vivo studies and scores as calculated from the ToxRTool evaluation [62].

Refs.	Type of Study	Species Used	Purity	Route	Dose Tested (µg/kg bw)	LD50 (µg/kg bw)	NOAEL µg/kg bw	Quality Score
[67]	Acute	SWISS mouse (female)	98%	Gavage	25, 75, 125, 250, 500, and 1000	232	75 *	2
[71]	Acute	SWISS mouse (female)	97%	Gavage	25, 75, 125, 250, 500, and 1000	Not indicated		2
[69]	Acute	SWISS mouse (female)	99.54%	Feeding (cream cheese)	360, 400, 450, 800, 900, and 1010	~900	~413	3
				Gavage	476, 533, 600, and 673	~600	ND	
				I.p. injection	6, 8, 10, 11, 13, 14, 16, 18, 19, 21, 22, 24, and 26	~10	ND	
[70]	Acute ** (intoxication model)	ICR mouse (both sexes)	99%	Gavage	250–400	ND	ND	1
				I.m. injection	4.5–9	ND	ND	
	Acute			Gavage	250, 325, 400, 500, and 600	379	ND; effects also seen at lowest dose	
				I.m. injection	6, 7, 8, 9, and 10	8.6		
	TK			Gavage	300			
				I.m. injection	7			
[72]	Acute	Sprague–Dawley rats with induced postherpetic neuralgia	98.5%	Feeding (pellet)	425, 500, 588, 692, and 814	Male/female 574/441	ND	1
	TK	Sprague–Dawley rats with induced postherpetic neuralgia		I.v. injection	6			
				Feeding (pellet)	100			
[73]	TK	Sprague–Dawley rats	98.3%	I.m. injection	6		ND	3
[74]	Repeated dose (28 days)	SWISS female mouse	96%	Gavage	20 and 44		ND; effects also seen at lowest dose	3
[68]	Repeated dose (28 days)	SWISS female mouse	>97%	Gavage	0, 25, 75, and 125		ND	3
[75]	Repeated dose ** (21 days)	SWISS mouse	98%	Feeding (cream cheese)	175/87.5, 250/125, and 325/162.5 (STX/TTX)		Values proposed (325 STX/162.5 TTX	3
[76]	Repeated dose (7 days)	SPF-grade ICR male mice	98%	Gavage	25, 75, and 125		ND	3
	TK				75			
[77]	Repeated dose (90 days)	Male albino rats	>97%	I.p. injection ***	0.5 and 1		ND	3–4
[78]	Repeated dose (90 days)	Male albino rats	>97%	I.p. injection ***	0.5 and 1		ND	3–4

* The indicated value is the NOAEL as derived by the authors. The EFSA uses as the point of departure the lower dose of 25 µg/kg bw. ** The endpoint evaluated was muscle strength by a grip strength test. *** The treatment frequency was set at weekly exposure.

**Table 2:** Relevant toxicological data extracted from the articles on TTX toxicity from in vitro studies and scores obtained from the ToxRTool evaluation [62].

Refs.	Cell Line Used	Purity	Administration	Measurement	Dose Tested (nM)	IC50 (nM)	Quality Score
[85]	Wistar rat neuronal cells and human iPSC astrocytes	98%	Direct in cell culture	Microelectrode array	1, 3, 10, and 30	7 (rat cells) 10 (human)	1
[86]	HEK293 transfect plasmid for Navs 1.1, 1.2, 1.3, 1.4, 1.5, 1.6, and 1.7	Not indicated	Direct in cell culture	Whole-cell patch clamp	0.3, 1, 3, 10, and 30 (higher for Na_V_ 1.5 and 1.7)	Nav 1.1: 4.1 Nav 1.2: 14 Nav 1.3: 5.3 Nav 1.4: 7.6 Nav 1.5: 1000 Nav 1.6: 2.3 Nav 1.7: 36	2
[87]	Wistar rat hyppocampal CA3 pyramidal neurons	Not indicated	Y-tube system in culture	Whole-cell patch clamp	0.1, 1, 3, 10, 100, 300, and 1000	37.9 for Ina^+^ current (hippocampal body)	2
[88]	Human iPSC cerebral organoid	Not indicated	Direct in organoid brain tissue	Tanscriptome analysis and cell viability	100, 1000, and 10,000	ND	3
In Vitro Toxicokinetic							
[83]	LLC-PK1 cell line	Not indicated	Direct in cell culture	LC-MS/MS and TEER (transepithelial electrical resistance)	50,000		2

**Table 3:** TTX occurrence in European bivalves and gastropods.

Sampling		LOQ (LOD) (μg TTX/kg)	Number of Samples	Species Analyzed	Sample > LOQ (Sample > LOD)	Highest Concentration (μg TTX/kg)			Refs.
Country	Year of Sampling (Frequency)					Mussels	Oysters	Gastropods	
United Kingdom	2014–2016 (NA)	0.93 (0.28)	1222	Mussels, oysters, cockles, and clams	15 (57)	19.9 (January 2016)	253 (July 2015)		* [36]
United Kingdom	2019 (weekly)	0.8 (NA)	78	Oysters	57 (57)		242 (dg) (July 2019)		* [92]
United Kingdom	2016 (monthly)	0.8 (0.2)	3514	Mussels, pacific oysters, and clams	41 (85)	93 (June 2016)			[30]
	2016–2018 (monthly)		371		62 (65)		202 (July 2018)		
Italy	2015–2017 (monthly: May–July)	0.93 (0.28)	23	Mussels and clams	13 (13)	6.0 (May 2016)			* [93]
Italy	2017–2018 (weekly: May–June)	2 (0.2)	8	Mussels	3 (3)	541 (May 2015)			* [94]
Italy	2019 (weekly: May–July; then, monthly)	25 (8)	30	Mussels and oysters	10 (10)	276 (June 2019)	127 (June 2019)		[25]
Italy	2020–2021 (fortnightly)	8 (3)	138	Mussels	34 (34)	296 (June 2021)			[27]
Netherlands	2015–2017 (weekly: June–October)	20 (3)	1063	Mussels, oysters, cockles, and clams	31 (69)	101 (June 2016)	253 (June 2016)		* [20]
France	2017 (NA)	15.0 (3.8)	66	Whelks	1 (1)			<LOQ	* [32]
	2018 (monthly)		127	Shellfish	3 (3)	11.2 (July 2018)			
France	2018 (weekly: June–October)	5 (3.8)	35	Mussels, oysters, and clams	2 (11)		12.2 (July 2018)		* [34]
	2019 (fortnightly: May–September)	12.5 (6.3)	63		1 (8)		32 (June 2019)		
France	2019–2022 (monthly)	30 (11)	436	Mussels, oysters, clams, and welks	0 (0)	<LOQ	<LOQ	<LOQ	[95]
France	2021 (fortnightly: May, Sept–Oct; weekly: June–July)	12.5 (5)	13	Oysters	3 (4)		424 (dg) (June 2021)		[28]
Spain	2017 (weekly: January–September)	0.9 (NA)	286	Mussels, oysters, cockles, clams, scallops, and razor clams	0 (2)	<LOD	<0.9		* [33]
Spain	2018–2019 (sporadic: June 2018–March 2019)	10 (NA)	47	Mussels, oysters, and gastropods	0 (0)	<LOQ	<LOQ	<LOQ	* [29]
Portugal	2015 (sporadic: April–September)	0.5 ng/mL (0.17) ng/mL	20	Clams (*R. decussatus*; *R. philippinarum*)	0 (0)	<LOQ			[96]
Portugal	2017 (obtained from market)	0.9 (0.3)	3	Gastropods (*Charonia lampas*)	0 (3)			42,160 (dg) 31.3 (et)	[97]
Portugal	2018 (weekly: May–October)	16 (NA)	120	Mussels, clams, and oysters	0 (0)	<LOQ	<LOQ		[35]
Portugal	Not indicated (obtained from market)	0.42–6.29 (0.13–1.89)	3	Gastropods (*Charonia lampas*)	3 (3)			7147.1 (ne) 966.3 (et)	[98]

* Cited in the review by [22].

**Table 4:** TTX occurrence in fish from the Tetraodontidae family in the Mediterranean Sea.

Sampling		Technique Used	No. of Samples (n)	Highest Concentration (μg TTX/g) *						Refs.
Country	Year of Sampling			Liver	Gonads	Skin	Intestines	Kidneys	Muscles	
Spain	2013–2014	HPLC MS/MS	1 (F)	4.6–5.36	20.0–25.22	1.8–2.08	not tested	not tested	0.9–0.98	[99]
Italy	2020–2021	HPLC MS/MS	20	<LOQ	<LOQ	<LOQ	<LOQ	not tested	<LOQ	[100]
Croatia	2014	HPLC MS/MS		30.6	48.7	1.5	not tested	not tested	0.8	[101]
Greece	2007	HPLC CID MS/MS	6 (NS) **	<LOQ-44.15	0.47–46.30	<LOQ-1.4	<LOQ-37.60	not tested	<LOQ-3.47	[102]
Greece	2017	HPLC Orbitrap MS/MS	2 (I)	0.733 *	0.733 *	1.188–1.239	not tested	not tested	0.478–2.077	[103]
Greece	2017	HPLC MS/MS	37	1.1–239.8	1.2–85.2	0.3–27.5	not tested	not tested	0.2–21.7	[104]
Greece (Crete)	2017–2020	HPLC MS/MS	83	0.04–104.41 (0.09–312.95)	0.30–189.03 (0.43–535.78)	0.12–18.59 (0.17–35.05)	not tested	not tested	0.02–20.72 (0.05–41.47)	[105]
Turkey	2012–2013	HPLC MS/MS	14 (M) **	25.4	2.58	3.3	48.8	34	0.342	[106]
			4 (F) **	2.18	80	0.5	0.62	1.77	0.29	
Turkey	2012–2013	HPLC MS/MS	12 (M)	<LOQ-0.40	0.43–30.70	0.13–1.31	0.10–0.92	not tested	<LOQ-0.44	[107]
			12 (F)	0.13–46.18	0.58–52.07	0.60–3.43	0.07–7.64	not tested	<LOQ-2.83	
Turkey	2015–2016	Q-TOF LC/MS	120 (M)	1.7–12.3	0.69–11.8	2.-9–5.02	2.29–3	not tested	1.7–12.3	[108]
			120 (F)	0.89–21.1	4.15–35.6	2.2–11.8	0.79–12.5	not tested	0.7–5.12	
Turkey	2015–2016	Q-TOF LC/MS	40 (M)	7.04–85.63	5.03–61.05	33.95–139.88	14.89–86.30	not tested	15.88–86.07	[109]
			40 (F)	11.62–106.8	41.49–100.71	35.19–139.72	12.59–55.24	not tested	42.07–72.38	
Turkey	2018–2019	Q-TOF LC/MS	55 (M)	5.8–21.6	1.4–68.2	not tested	not tested	not tested	1.3–6.0	[110]
			55 (F)	0.8–34.2	6.8–65.9	not tested	not tested	not tested	1.3–7.8	
Cyprus	2017–2018	ELISA	5 (M)	0.18–13.48	0.38–6.84	0.16–1.78	0.52–1.48	not tested	0.22–3	[111]
			11 (F)	0.11–13.33	0.32–12.87	0.24–6.54	0.29–11.74	not tested	0.21–8.34	
Lebanon	Winter 2020	HPLC MS/MS	1 (M)	12.96	0.26	0.17	not tested	not tested	0.26	[112]
			2 (F)	46.67	252.97	0.71	not tested	not tested	0.6	

* It is worth noting the different levels of concentration with respect to mollusks, in μg/g vs. μg/Kg. ** M = male; F = female; NS = not specified.

## Data Availability

No new data were created.

## Supplementary Materials

The following supporting information can be downloaded at https://www.mdpi.com/article/10.3390/toxins17020076/s1. Figure S1: Prisma graph representing the search process for occurrence in edible seafoo; Figure S2: Prisma graph representing the search process for occurrence in pufferfish; Table S1: Objectives, database used, and queries applied for the extensive literature search; Table S2: Data from FoodEx2.

## Registration and Protocol

This review was not registered and a protocol was not prepared. We followed the procedure extensively described in Section 2 (Methodology) and in the Supplementary Material.

## References

[1] EC (2004). Regulation (EC) No 853/2004 of the European Parliament and of the Council of 29 April 2004 laying down specific hygiene rules for food of animal origin. Off. J. Eur. Union.

[2] EC (2017). Regulation (EU) 2017/625 of the uropean parliament and of the council of 15 March 2017 on official controls and other official activities performed to ensure the application of food and feed law, rules on animal health and welfare, plant health an plant protection products, amending regulations (EC) No 999/2001, (EC) 396/2205. (EC) No 1069/2009, (EC) No 1107/2009, (EU) No 1151/2012, (EC) No 625/2014, (EU) 2016/429 and (EU) 2016/2031 of the European Parliament and of the Council, Council regulations (EC) No 1/2005 and (EC) No1099/2009, and Council Directives 98/58/EC, 1999/74/EC, 2007/43/EC, 2008/119/EC and 2008/120/EC, and repealing reguòatiom (EC) No 854/2004 and (EC) No 882/2004 of the European Parliament and of the Council, Council Directives 89/608/EEC, 89/662/EEC, 90/425/EEC, 91/496/EEC, 96/23/EEC, 96/93/EC and /97/98/EC and Council Decision 92/438/EEC (official controls regulation). Off. J. Eur. Union L.

[3] EC (2019). Commission implementing regulation (EU) 2019/627 of 15 March 2019 laying down uniform practical arrangements for the performance of official controls on products of animal origin intended for uhman consumption in accordance with Regulation (EU) 2017/625 of the European Parliament and of the Council and amending Commission Regulation (EC) No 2074/2005 as regards official controls. Off. J. Eur. Union.

[4] Turner, Powell A., Schofield A., Lees D.N., Baker-Austin C. (2015). Detection of the pufferfish toxin tetrodotoxin in European bivalves, England, 2013 to 2014. Euro Surveill.

[5] Vlamis A., Katikou P., Rodriguez I., Rey V., Alfonso A., Papazachariou A., Zacharaki T., Botana A.M., Botana L.M. (2015). First detection of tetrodotoxin in Greek shellfish by UPLC-MS/MS potentially linked to the presence of the dinoflagellate Prorocentrum minimum. Toxins.

[6] Williams B.L. (2010). Behavioral and Chemical Ecology of Marine Organisms with Respect to Tetrodotoxin. Mar. Drugs.

[7] Guardone L., Maneschi A., Meucci V., Gasperetti L., Nucera D., Armani A. (2020). A Global Retrospective Study on Human Cases of Tetrodotoxin (TTX) Poisoning after Seafood Consumption. Food Rev. Int..

[8] Ministry of Health Labour Welfare of Japan (2019). Pufferfish Human Poisoning 19 2003–2017. https://www.mhlw.go.jp/topics/syokuchu/poison/dl/animal_det_01-02.pdf.

[9] Por F.D. (1978). Lessepsian Migration: The Influx of Red Sea Biota into the Mediterranean by Way of the Suez Canal.

[10] Hallegraeff G., Enevoldsen H., Zingone A. (2021). Global harmful algal bloom status reporting. Harmful Algae.

[11] GlobalHAB Scientific Committee (2021). Guidelines for the Study of Climate Change Effects on HABs.

[12] Heisler J., Glibert P.M., Burkholder J.M., Anderson D.M., Cochlan W., Dennison W.C., Dortch Q., Gobler C.J., Heil C.A., Humphries E. (2008). Eutrophication and harmful algal blooms: A scientific consensus. Harmful Algae.

[13] Diogène J., Rambla M., Campàs M., Fernández M., Andree K., Tudó A., Rey M., Sagristà N., Aguayo P., Leonardo S. (2021). Evaluation of ciguatoxins in seafood and the environment in Europe. EFSA Support. Publ..

[14] Maggiore A., Afonso A., Barrucci F., Sanctis G.D., EFSA (2020). Climate change as a driver of emerging risks for food and feed safety, plant, animal health and nutritional quality. EFSA Support. Publ..

[15] Rodríguez P., Alfonso A., Vale C., Alfonso C., Vale P., Tellez A., Botana L.M. (2008). First Toxicity Report of Tetrodotoxin and 5,6,11-TrideoxyTTX in the Trumpet Shell Charonia lampas lampas in Europe. Anal. Chem..

[16] Knutsen H.K., Alexander J., Barregård L., Bignami M., Brüschweiler B., Ceccatelli S., Cottrill B., Dinovi M., Edler L., EFSA CONTAM Panel (EFSA Panel on Contaminants in the Food Chain) (2017). Risks for public health related to the presence of tetrodotoxin (TTX) and TTX analogues in marine bivalves and gastropods. EFSA J..

[17] Biessy L., Boundy M.J., Smith K.F., Harwood D.T., Hawes I., Wood S.A. (2019). Tetrodotoxin in marine bivalves and edible gastropods: A mini-review. Chemosphere.

[18] EFSA Panel on Contaminants in the Food Chain (CONTAM) (2010). Statement on further elaboration of the consumption figure of 400 g shellfish meat on the basis of new consumption data. EFSA J..

[19] Mi W., Liu S. (2024). Tetrodotoxin and the state-of-the-art progress of its associated analytical methods. Front. Microbiol..

[20] Gerssen A., Bovee T., Klijnstra M., Poelman M., Portier L., Hoogenboom R. (2018). First Report on the Occurrence of Tetrodotoxins in Bivalve Mollusks in The Netherlands. Toxins.

[21] Sinno-Tellier S., Abadie E., Guillotin S., Bossée A., Nicolas M., Delcourt N. (2023). Human shellfish poisoning: Implementation of a national surveillance program in France. Front. Mar. Sci..

[22] Antonelli P., Salerno B., Bordin P., Peruzzo A. (2022). Tetrodotoxin in live bivalve mollusks from Europe: Is it to be considered an emerging concern for food safety?. Comp. Rev. Food. Sci. Food. Safe.

[23] Katikou P. (2019). Public Health Risks Associated with Tetrodotoxin and Its Analogues in European Waters: Recent Advances after The EFSA Scientific Opinion. Toxins.

[24] Katikou P., Gokbulut C., Kosker A.R., Campàs M., Ozogul F. (2022). An Updated Review of Tetrodotoxin and Its Peculiarities. Mar. Drugs.

[25] Antonelli P., Peruzzo A., Mancin M., Boscolo Anzoletti A., Dall’Ara S., Orsini M., Bordin P., Arcangeli G., Zanolin B., Barco L. (2023). Tetrodotoxin in bivalve mollusks: An integrated study towards the comprehension of the influencing factors of a newly native phenomenon. Chemosphere.

[26] Bacchiocchi S., Campacci D., Siracusa M., Dubbini A., Leoni F., Tavoloni T., Accoroni S., Gorbi S., Giuliani M.E., Stramenga A. (2021). Tetrodotoxins (TTXs) and Vibrio alginolyticus in mussels from central Adriatic Sea (Italy): Are they closely related?. Mar. Drugs.

[27] Bacchiocchi S., Campacci D., Siracusa M., Dubbini A., Accoroni S., Romagnoli T., Campanelli A., Griffoni F., Tavoloni T., Gorbi S. (2023). A Hotspot of TTX Contamination in the Adriatic Sea: Study on the Origin and Causative Factors. Mar. Drugs.

[28] Biessy L., Pearman J.K., Mertens K.N., Réveillon D., Savar V., Hess P., Hampton H., Thompson L., Lebrun L., Terre-Terrillon A. (2024). Sudden peak in tetrodotoxin in French oysters during the summer of 2021: Source investigation using microscopy, metabarcoding and droplet digital PCR. Toxicon.

[29] Blanco L., Lago J., González V., Paz B., Rambla-Alegre M., Cabado A.G. (2019). Occurrence of tetrodotoxin in bivalves and gastropods from harvesting areas and other natural spaces in Spain. Toxins.

[30] Dhanji-Rapkova M., Teixeira Alves M., Triñanes J.A., Martinez-Urtaza J., Haverson D., Bradley K., Baker-Austin C., Huggett J.F., Stewart G., Ritchie J.M. (2023). Sea temperature influences accumulation of tetrodotoxin in British bivalve shellfish. Sci. Total Environ..

[31] Dhanji-Rapkova M., Hatfield R.G., Walker D.I., Hooper C., Alewijnse S., Baker-Austin C., Turner A.D., Ritchie J.M. (2024). Investigating Non-Native Ribbon Worm Cephalothrix simula as a Potential Source of Tetrodotoxin in British Bivalve Shellfish. Mar. Drugs.

[32] Hort V., Arnich N., Guérin T., Lavison-Bompard G., Nicolas M. (2020). First Detection of Tetrodotoxin in Bivalves and Gastropods from the French Mainland Coasts. Toxins.

[33] Leão J.M., Lozano-Leon A., Giráldez J., Vilariño Ó., Gago-Martínez A. (2018). Preliminary results on the evaluation of the occurrence of tetrodotoxin associated to marine Vibrio spp. in bivalves from the Galician Rias (Northwest of Spain). Mar. Drugs.

[34] Réveillon D., Savar V., Schaefer E., Chevé J., Halm-Lemeille M.-P., Hervio-Heath D., Travers M.-A., Abadie E., Rolland J.-L., Hess P. (2021). Tetrodotoxins in French Bivalve Mollusks—Analytical Methodology, Environmental Dynamics and Screening of Bacterial Strain Collections. Toxins.

[35] Rodrigues S.M., Pinto E.P., Oliveira P., Pedro S., Costa P.R. (2019). Evaluation of the Occurrence of Tetrodotoxin in Bivalve Mollusks from the Portuguese Coast. J. Mar. Sci. Eng..

[36] Turner A.D., Dhanji-Rapkova M., Coates L., Bickerstaff L., Milligan S., O’Neill A., Faulkner D., McEneny H., Baker-Austin C., Lees D.N. (2017). Detection of Tetrodotoxin Shellfish Poisoning (TSP) toxins and causative factors in bivalve molluscs from the UK. Mar. Drugs.

[37] Bille L., Binato G., Cappa V., Toson M., Dalla Pozza M., Arcangeli G., Ricci A., Angeletti R., Piro R. (2015). Lead, mercury and cadmium levels in edible marine molluscs and echinoderms from the Veneto Region (north-western Adriatic Sea—Italy). Food Control.

[38] FAO (2024). The State Of. World Fisheries and Aquaculture 2024—Bue Transformation in Action.

[39] Saoudi M., Rabeh F.B., Jammoussi K., Abdelmouleh A., Belbahri L., El Feki A. (2007). Biochemical and physiological responses in Wistar rat after administration of puffer fish (Lagocephalus lagocephalus) flesh. J. Food. Agric. Environ..

[40] Magarlamov T., Melnikova D., Chernyshev A. (2017). Tetrodotoxin-Producing Bacteria: Detection, Distribution and Migration of the Toxin in Aquatic Systems. Toxins.

[41] Melnikova D.I., Vlasenko A.E., Magarlamov T.Y. (2019). Stable tetrodotoxin production by *Bacillus* sp. Strain 1839. Mar. Drugs.

[42] Kodama M., Sato S., Sakamoto S., Ogata T. (1996). Occurrence of tetrodotoxin in Alexandrium tamarense, a causative dinoflagellate of paralytic shellfish poisoning. Toxicon.

[43] Grzebyk D., Denardou A., Berland B., Pouchus Y.F. (1997). Evidence of a new toxin in the red-tide dinoflagellate *Prorocentrum Minimum*. J. Plankton Res..

[44] John U., Tillmann U., Hülskötter J., Alpermann T.J., Wohlrab S., Van De Waal D.B. (2015). Intraspecific facilitation by allelochemical mediated grazing protection within a toxigenic dinoflagellate population. Proc. R. Soc. B.

[45] Noguchi T., Arakawa O. (2008). Tetrodotoxin—Distribution and accumulation in aquatic organisms, and cases of human intoxication. Mar. Drugs.

[46] Roy M., Narahashi T. (1992). Differential properties of tetrodotoxin-sensitive and tetrodotoxin- resistant sodium channels in rat dorsal root ganglion neurons. J. Neurosci..

[47] Bane V., Lehane M., Dikshit M., O’Riordan A., Furey A. (2014). Tetrodotoxin: Chemistry, toxicity, source, distribution and detection. Toxins.

[48] Noreng S., Li T., Payandeh J. (2021). Structural Pharmacology of Voltage-Gated Sodium Channels. J. Mol. Biol..

[49] Fukuda A., Tani A. (1941). Records of pufferfish poisonings. Report 3. Nippon. Igaku Oyobi Kenko.

[50] Lago J., Rodríguez L., Blanco L., Vieites J., Cabado A. (2015). Tetrodotoxin, an Extremely Potent Marine Neurotoxin: Distribution, Toxicity, Origin and Therapeutical Uses. Mar. Drugs.

[51] Rosker C., Lohberger B., Hofer D., Steinecker B., Quasthoff S., Schreibmayer W. (2007). The TTX metabolite 4,9-anhydro-TTX is a highly specific blocker of the Na_v1.6_ voltage-dependent sodium channel. Am. J. Physiol.—Cell Physiol..

[52] Lee C.H., Ruben P.C. (2008). Interaction between voltage-gated sodium channels and the neurotoxin, tetrodotoxin. Channels.

[53] Catterall W.A., Goldin A.L., Waxman S.G. (2005). International Union of Pharmacology. XLVII. Nomenclature and Structure-Function Relationships of Voltage-Gated Sodium Channels. Pharmacol. Rev..

[54] Isbister G.K., Kiernan M.C., Lin C.S.Y., Burke D., Bostock H. (2005). Acute tetrodotoxin-induced neurotoxicity after ingestion of puffer fish. Ann. Neurol..

[55] Zimmer T. (2010). Effects of Tetrodotoxin on the Mammalian Cardiovascular System. Mar. Drugs.

[56] Soong T., Venkatesh B. (2006). Adaptive evolution of tetrodotoxin resistance in animals. Trends. Genet..

[57] Choudhary G., Yotsu-Yamashita M., Shang L., Yasumoto T., Dudley S.C. (2003). Interactions of the C-11 Hydroxyl of Tetrodotoxin with the Sodium Channel Outer Vestibule. Biophys. J..

[58] Teramoto N., Yotsu-Yamashita M. (2015). Selective Blocking Effects of 4,9-Anhydrotetrodotoxin, Purified from a Crude Mixture of Tetrodotoxin Analogues, on NaV1.6 Channels and Its Chemical Aspects. Mar. Drugs.

[59] Chen R., Chung S.-H. (2014). Mechanism of tetrodotoxin block and resistance in sodium channels. Biochem. Biophys. Res. Commun..

[60] EFSA (2010). Application of systematic review methodology to food and feed safety assessments to support decision making. EFSA J..

[61] Klimisch H.J., Andreae M., Tillmann U. (1997). A Systematic Approach for Evaluating the Quality of Experimental Toxicological and Ecotoxicological Data. Regul. Toxicol. Pharm..

[62] Schneider K., Schwarz M., Burkholder I., Kopp-Schneider A., Edler L., Kinsner-Ovaskainen A., Hartung T., Hoffmann S. (2009). “ToxRTool”, a new tool to assess the reliability of toxicological data. Toxicol. Lett..

[63] Testai E., Buratti F.M., Funari E., Manganelli M., Vichi S., Arnich N., Biré R., Fessard V., Sialehaamoa A. (2016). Review and analysis of occurrence, exposure and toxicity of cyanobacteria toxins in food. EFSA Support. Publ..

[64] WHO (2021). WHO Human Health Risk Assessment Toolkit: Chemical Hazards.

[65] Kavoosi M., O’Reilly T.E., Kavoosi M., Chai P., Engel C., Korz W., Gallen C.C., Lester R.M. (2020). Safety, Tolerability, Pharmacokinetics, and Concentration-QTc Analysis of Tetrodotoxin: A Randomized, Dose Escalation Study in Healthy Adults. Toxins.

[66] Chen W., Zhang Y., Fang H., Chen H., He J., Yi R., Hong Z. (2020). Development and validation of a specific and sensitive liquid chromatography tandem mass spectrometry method for determination of tetrodotoxin in human urine and its pharmacokinetic study. Biomed. Chromatogr..

[67] Abal P., Louzao M., Antelo A., Alvarez M., Cagide E., Vilariño N., Vieytes M., Botana L. (2017). Acute Oral Toxicity of Tetrodotoxin in Mice: Determination of Lethal Dose 50 (LD50) and No Observed Adverse Effect Level (NOAEL). Toxins.

[68] Boente-Juncal A., Vale C., Cifuentes M., Otero P., Camiña M., Rodriguez-Vieytes M., Botana L.M. (2019). Chronic In Vivo Effects of Repeated Exposure to Low Oral Doses of Tetrodotoxin: Preliminary Evidence of Nephrotoxicity and Cardiotoxicity. Toxins.

[69] Finch S., Boundy M., Harwood D. (2018). The Acute Toxicity of Tetrodotoxin and Tetrodotoxin–Saxitoxin Mixtures to Mice by Various Routes of Administration. Toxins.

[70] Wang F., Zhang F., Song J., Zou S., Li J., Huang Y., Zhang L., Wang Q. (2023). Acute Toxic Effects of Tetrodotoxin in Mice via Intramuscular Injection and Oral Gavage. Toxins.

[71] Abal P., Louzao M.C., Vilariño N., Vieytes M.R., Botana L.M. (2019). Acute Toxicity Assessment: Macroscopic and Ultrastructural Effects in Mice Treated with Oral Tetrodotoxin. Toxins.

[72] Bihong H., Sun J., Zheng H., Le Q., Wang C., Bai K., He J., He H., Dong Y. (2018). Effect of Tetrodotoxin Pellets in a Rat Model of Postherpetic Neuralgia. Mar. Drugs.

[73] Bihong H., Chen H., Han J., Xie Q., He J., Bai K., Dong Y., Yi R. (2017). A Study of 11-[3H]-Tetrodotoxin Absorption, Distribution, Metabolism and Excretion (ADME) in Adult Sprague-Dawley Rats. Mar. Drugs.

[74] Boente-Juncal A., Otero P., Rodríguez I., Camiña M., Rodriguez-Vieytes M., Vale C., Botana L.M. (2020). Oral chronic toxicity of the safe tetrodotoxin dose proposed by the European food safety authority and its additive effect with saxitoxin. Toxins.

[75] Finch S.C., Webb N.G., Boundy M.J., Harwood D.T., Munday J.S., Sprosen J.M., Somchit C., Broadhurst R.B. (2023). A Sub-Acute Dosing Study of Saxitoxin and Tetrodotoxin Mixtures in Mice Suggests That the Current Paralytic Shellfish Toxin Regulatory Limit Is Fit for Purpose. Toxins.

[76] Zhong Y., Zhang X., Yang Q., Wang Q. (2023). Hepatorenal Toxicity after 7-Day Oral Administration of Low-Dose Tetrodotoxin and Its Distribution in the Main Tissues in Mice. Toxins.

[77] Humadai T.J., Al-Kaisei B.I. (2024). Potential Pathological Effects After Repeated Exposure to Tetrodotoxin in Reproductive System of Albino Male Rats. Adv. Anim. Vet. Sci..

[78] Humadai T.J., Al-Kaisei B.I. (2024). Study The Pathological, Immunological Effects After Repeated Exposure to Tetrodotoxin in Liver of Albino Male Rats. Adv. Anim. Vet. Sci..

[79] Xu Q., Huang K., Gao L., Zhang H., Rong K. (2003). Toxicity of tetrodotoxin towards mice and rabbits. Wei Sheng Yan Jiu.

[80] Marcil J., Walczak J.S., Guindon J., Ngoc A.H., Lu S., Beaulieu P. (2006). Antinociceptive effects of tetrodotoxin (TTX) in rodents. Br. J. Anaesth..

[81] OECD (2022). Test No. 425: Acute Oral Toxicity: Up-and-Down Procedure. OECD Guidelines for the Testing of Chemicals, Section 4.

[82] OECD (2008). Test No. 407: Repeated Dose 28-day Oral Toxicity Study in Rodents. OECD Guidelines for the Testing of Chemicals, Section 4.

[83] Matsumoto T., Ishizaki Y., Mochizuki K., Aoyagi M., Mitoma Y., Ishizaki S., Nagashima Y. (2017). Urinary Excretion of Tetrodotoxin Modeled in a Porcine Renal Proximal Tubule Epithelial Cell Line, LLC-PK1. Mar. Drugs.

[84] Noorlander A., Zhang M., Van Ravenzwaay B., Rietjens I.M.C.M. (2022). Use of Physiologically Based Kinetic Modeling-Facilitated Reverse Dosimetry to Predict *In Vivo* Acute Toxicity of Tetrodotoxin in Rodents. Toxicol. Sci..

[85] Kasteel E.E.J., Westerink R.H.S. (2017). Comparison of the acute inhibitory effects of Tetrodotoxin (TTX) in rat and human neuronal networks for risk assessment purposes. Toxicol. Lett..

[86] Tsukamoto T., Chiba Y., Wakamori M., Yamada T., Tsunogae S., Cho Y., Sakakibara R., Imazu T., Tokoro S., Satake Y. (2017). Differential binding of tetrodotoxin and its derivatives to voltage-sensitive sodium channel subtypes (Na _v_ 1.1 to Na _v_ 1.7). Br. J. Pharmacol..

[87] Wakita M., Kotani N., Akaike N. (2015). Tetrodotoxin abruptly blocks excitatory neurotransmission in mammalian CNS. Toxicon.

[88] Liu Z., Wang Z., Wei Y., Shi J., Shi T., Chen X., Li L. (2023). Transcriptomic Profiling of Tetrodotoxin-Induced Neurotoxicity in Human Cerebral Organoids. Mar. Drugs.

[89] Al Homsi A., Hassan N., Hamad I., Qasem R. (2023). A Case of Puffer Fish Poisoning from United Arab Emirates. Oman Med. J..

[90] Al-Sulaimani S., Titelbaum N.V., Ward R.E., Zahran T.E., Chalhoub S., Kazzi Z. (2022). Case Report of Tetrodotoxin Poisoning from *Lagocephalus sceleratus* in Lebanon. Int. J. Environ. Res. Public. Health.

[91] Cheung K.S., Chan C.K. (2023). A 12-year retrospective review of tetrodotoxin poisoning in Hong Kong. Hong Kong J. Emerg. Med..

[92] Dhanji-Rapkova M., Turner A.D., Baker-Austin C., Huggett J.F., Ritchie J.M. (2021). Distribution of Tetrodotoxin in Pacific Oysters (*Crassostrea gigas*). Mar. Drugs.

[93] Dell’Aversano C., Tartaglione L., Polito G., Dean K., Giacobbe M., Casabianca S., Capellacci S., Penna A., Turner A.D. (2019). First detection of tetrodotoxin and high levels of paralytic shellfish poisoning toxins in shellfish from Sicily (Italy) by three different analytical methods. Chemosphere.

[94] Bordin P., Dall’Ara S., Tartaglione L., Antonelli P., Calfapietra A., Varriale F., Guiatti D., Milandri A., Dell’Aversano C., Arcangeli G. (2021). First occurrence of tetrodotoxins in bivalve mollusks from Northern Adriatic Sea (Italy). Food Control.

[95] Amzil Z., Derrien A., Terre Terrillon A., Savar V., Bertin T., Peyrat M., Duval A., Lhaute K., Arnich N., Hort V. (2023). Five Years Monitoring the Emergence of Unregulated Toxins in Shellfish in France (EMERGTOX 2018–2022). Mar. Drugs.

[96] Braga A.C., Lage S., Pacheco M., Rydberg S., Costa P.R. (2017). Native (*Ruditapes decussatus*) and non-indigenous (*R. philippinarum*) shellfish species living in sympatry: Comparison of regulated and non-regulated biotoxins accumulation. Mar. Environ. Res..

[97] Costa P.R., Giráldez J., Rodrigues S.M., Leão J.M., Pinto E., Soliño L., Gago-Martínez A. (2021). High Levels of Tetrodotoxin (TTX) in Trumpet Shell Charonia lampas from the Portuguese Coast. Toxins.

[98] Lage S., Afonso I.I., Reis Costa P., Canário A.V.M., Da Silva J.P. (2023). Tissue accumulation of tetrodotoxin (TTX) and analogues in trumpet shell *Charonia lampas*. Food Addit. Contam..

[99] Rambla-Alegre M., Reverté L., del Río V., de la Iglesia P., Palacios O., Flores C., Caixach J., Campbell K., Elliott C.T., Izquierdo-Muñoz A. (2017). Evaluation of tetrodotoxins in puffer fish caught along the Mediterranean coast of Spain. Toxin profile of *Lagocephalus sceleratus*. Environ. Res..

[100] Malloggi C., Rizzo B., Giusti A., Guardone L., Gasperetti L., Dall’Ara S., Armani A. (2023). First Toxicological Analysis of the Pufferfish Sphoeroides pachygaster Collected in Italian Waters (Strait of Sicily): Role of Citizens Science in Monitoring Toxic Marine Species. Animals.

[101] Ujević I., Roje-Busatto R., Dragičević B., Dulčić J. (2020). Tetrodotoxin in Invasive Silver-cheeked Toadfish *Lagocephalus sceleratus* (Gmelin, 1789) in the Adriatic Sea. Handb. Environ. Chem.

[102] Rodríguez P., Alfonso A., Otero P., Katikou P., Georgantelis D., Botana L.M. (2012). Liquid chromatography–mass spectrometry method to detect Tetrodotoxin and Its analogues in the puffer fish *Lagocephalus sceleratus* (Gmelin, 1789) from European waters. Food Chem..

[103] Leonardo S., Kiparissis S., Rambla-Alegre M., Almarza S., Roque A., Andree K.B., Christidis A., Flores C., Caixach J., Campbell K. (2019). Detection of tetrodotoxins in juvenile pufferfish *Lagocephalus sceleratus* (Gmelin, 1789) from the North Aegean Sea (Greece) by an electrochemical magnetic bead-based immunosensing tool. Food Chem..

[104] Anastasiou T.I., Kagiampaki E., Kondylatos G., Tselepides A., Peristeraki P., Mandalakis M. (2023). Assessing the Toxicity of *Lagocephalus sceleratus* Pufferfish from the Southeastern Aegean Sea and the Relationship of Tetrodotoxin with Gonadal Hormones. Mar. Drugs.

[105] Christidis G., Mandalakis M., Anastasiou T.I., Tserpes G., Peristeraki P., Somarakis S. (2021). Keeping *Lagocephalus sceleratus* off the Table: Sources of Variation in the Quantity of TTX, TTX Analogues, and Risk of Tetrodotoxication. Toxins.

[106] Acar C., Ishizaki S., Nagashima Y. (2017). Toxicity of the Lessepsian pufferfish *Lagocephalus sceleratus* from eastern Mediterranean coasts of Turkey and species identification by rapid PCR amplification. Food Sci. Technol. Res..

[107] Kosker A.R., Özogul F., Durmus M., Ucar Y., Ayas D., Regenstein J.M., Özogul Y. (2016). Tetrodotoxin levels in pufferfish (*Lagocephalus sceleratus*) caught in the Northeastern Mediterranean Sea. Food Chem..

[108] Kosker A.R., Özogul F., Ayas D., Durmus M., Ucar Y., Regenstein J.M., Özogul Y. (2019). Tetrodotoxin levels of three pufferfish species (*Lagocephalus* sp.) caught in the North-Eastern Mediterranean sea. Chemosphere.

[109] Kosker A.R., Özogul F., Durmus M., Ucar Y., Ayas D., Šimat V., Özogul Y. (2018). First report on TTX levels of the yellow spotted pufferfish (*Torquigener flavimaculosus*) in the Mediterranean Sea. Toxicon.

[110] Kosker A., Karakus M., Katikou P., Dal İ., Durmus M., Ucar Y., Ayas D., Özogul F. (2023). Monthly Variation of Tetrodotoxin Levels in Pufferfish (*Lagocephalus sceleratus*) Caught from Antalya Bay, Mediterranean Sea. Mar. Drugs.

[111] Akbora H.D., Kunter İ., Erçetïn T., Elagöz A.M., Çïçek B.A. (2020). Determination of tetrodotoxin (TTX) levels in various tissues of the silver cheeked puffer fish (*Lagocephalus sceleratus* (Gmelin, 1789)) in Northern Cyprus Sea (Eastern Mediterranean). Toxicon.

[112] Hassoun A.E.R., Ujević I., Jemaa S., Roje-Busatto R., Mahfouz C., Fakhri M., Nazlić N. (2022). Concentrations of Tetrodotoxin (TTX) and Its Analogue 4,9-Anhydro TTX in Different Tissues of the Silver-Cheeked Pufferfish (*Lagocephalus sceleratus*, Gmelin, 1789) Caught in the South-Eastern Mediterranean Sea, Lebanon. Toxins.

[113] Nader M., Indary S., Boustany L. The Puffer Fish *Lagocephalus sceleratus* (Gmelin, 1789) in the Eastern Mediterranean. 2012. FAO EastMed Technical Document GCP/INT/041/EC—GRE—ITA/TD-10. https://openknowledge.fao.org/handle/20.500.14283/ap967e.

[114] Corsini-Foka M., Margies P., Kondilatos G., Economidis P.S. (2006). Torquigener flavimaculosus Hardy and Randall, 1983 (Pisces: Tetraodontidae) off Rhodes island marine area: A new alien fish in the Hellenic waters. Medit. Mar. Sci..

[115] Beköz A.B., Beköz S., Yilmaz E., Tüzün S., Beköz U. (2013). Consequences of the increasing prevalence of the poisonous Lagocephalus sceleratus in southern Turkey. Emerg. Med. J..

[116] Souissi J., Rifi M., Ghanem R., Ghozzi L., Boughedir W., Azzurro E. (2014). *Lagocephalus sceleratus* (Gmelin, 1789) expands through the African coasts towards the Western Mediterranean Sea: A call for awareness. Manag. Biol. Invasions.

[117] Deidun A., Fenech-Farrugia A., Castriota L., Falautano M., Azzurro E., Andaloro F. (2015). First record of the silver-cheeked toadfish *Lagocephalus sceleratus* (Gmelin, 1789) from Malta. BioInvasions Rec..

[118] Azzurro E., Castriota L., Falautano M., Giardina F., Andaloro F. (2014). The silver-cheeked toadfish *Lagocephalus sceleratus* (Gmelin, 1789) reaches Italian waters. J. Appl. Ichthyol..

[119] Azzurro E., Castriota L., Falautano M., Bariche M., Broglio E., Andaloro F. (2016). New records of the silver-cheeked toadfish *Lagocephalus sceleratus* (Gmelin, 1789) in the Tyrrhenian and Ionian Seas: Early detection and participatory monitoring in practice. BioInvasions Rec..

[120] Sulić Šprem J., Dobroslavić T., Kožul V., Kuzman A., Dulčić J. (2014). First record of *Lagocephalus sceleratus* in the Adriatic Sea (Croatian coast), a Lessepsian migrant. Cybium.

[121] Losasso C., Bille L., Patuzzi I., Lorenzetto M., Binato G., Dalla Pozza M., FerrÃ¨ N., Ricci A. (2015). Possible Influence of Natural Events on Heavy Metals Exposure from Shellfish Consumption: A Case Study in the North-East of Italy. Front. Public Health.

[122] Funari E., Manganelli M., Testai E. (2015). Ostreospis cf. ovata blooms in coastal water: Italian guidelines to assess and manage the risk associated to bathing waters and recreational activities. Harmful Algae.

