# Supplementary material for: An Update of Tetrodotoxins Toxicity and Risk Assessment Associated to Contaminated Seafood Consumption in Europe: A Systematic Review
Source: Toxins (Basel). 2025 Feb 8;17(2):76. doi: 10.3390/toxins17020076 (PMC11860457; doi:10.3390/toxins17020076)
Supplement: Supplementary file 1 [file toxins-17-00076-s001.zip › toxins-3430617-supplementary.pdf]

SUPPLEMENTARY MATERIAL

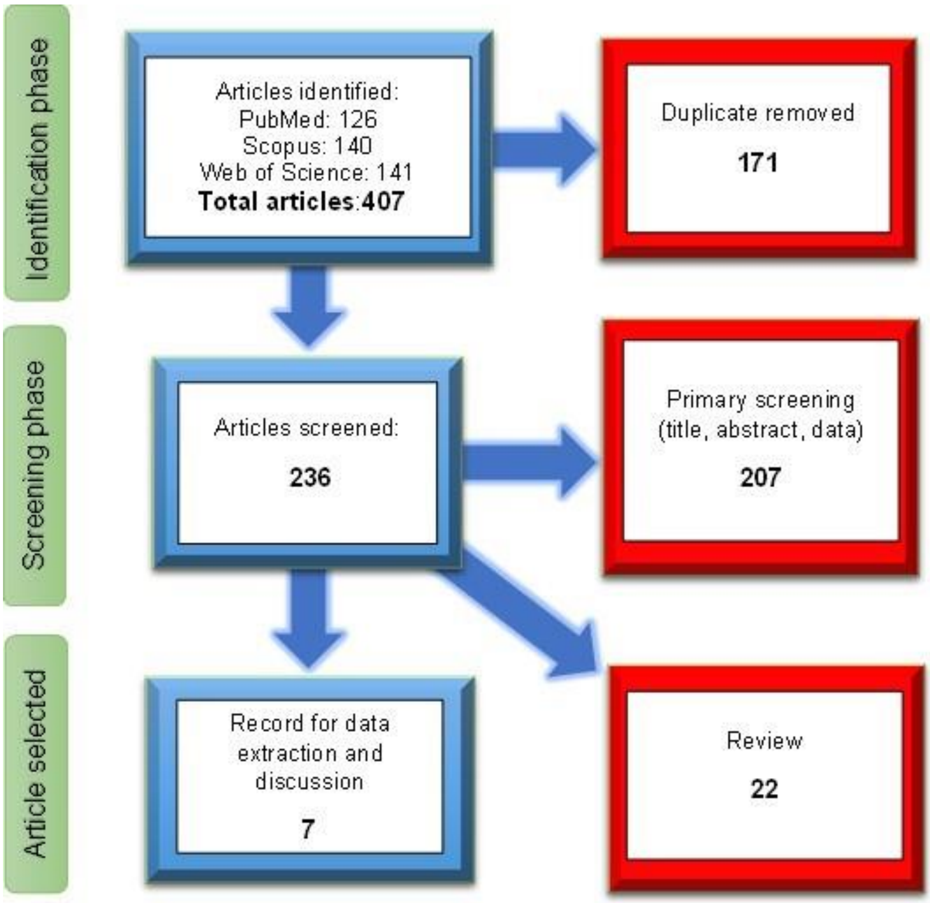

Figure S1 Prisma graph representing the search process for occurrence in edible seafood.

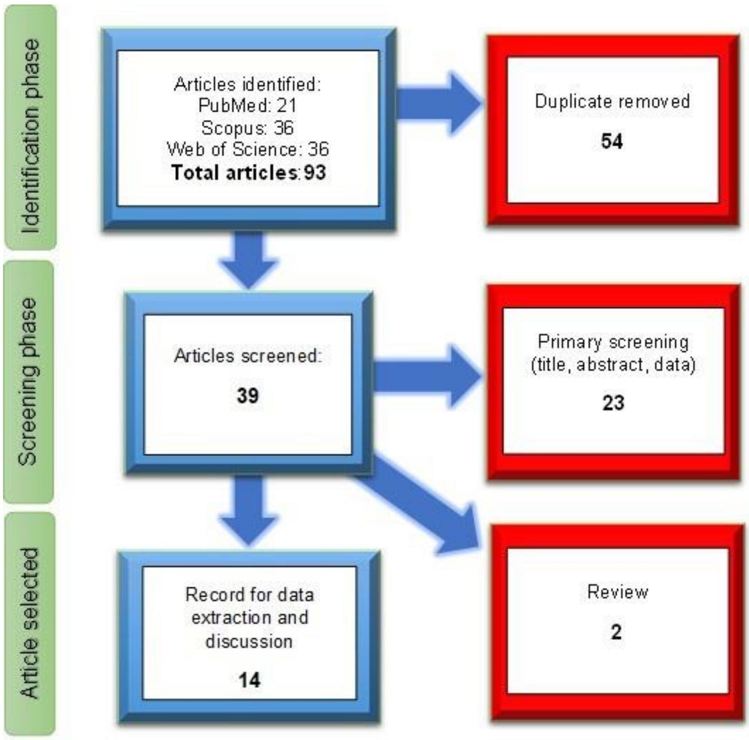

Figure S2 Prisma graph representing the search process for occurrence in pufferfish.

Table S1 Objectives, database used, and queries applied for the extensive literature search.

SYSTEMATIC REVIEW	EXTENSIVE LITERATURE SEARCH ON TTX TOXIC EFFECTS
**STUDY QUESTION**	**Objective 1:** To update the TTX hazard characterization<br>**Objective 2:** To update data on TTX occurrence in molluscs and other seafood in Europe.<br>**Objective 3:** data on TTX occurrence in pufferfish in Europe
**Database**	PubMed, Scopus and Science Direct
**Search terms Objective 1**	*Query Search PubMed*:((tetrodotoxin) **OR** (anhydrotetrodotoxin) **OR** (4-epitetrodotoxin) **OR** (tetrodonic acid)) **AND** ((toxic*) **OR** (toxicity) **OR** (health effects) **OR** neurotoxin*) **OR** (paralysis))<br><br>*Query Search Scopus 1*: ((tetrodotoxin) **OR** (anhydrotetrodotoxin) **OR** (4-epitetrodotoxin) **OR** (tetrodonic acid)) **AND** ((toxic*) **OR** (toxicity) **OR** (health effects) **OR** (neurotoxin*) **OR** (paralysis))<br>*Query Search Scopus 2*: **TITLE-ABS-KEY** ((tetrodotoxin) **OR** (anhydrotetrodotoxin) **OR** (4-epitetrodotoxin) **OR** (tetrodonic acid)) **AND TITLE-ABS-KEY** ((toxic*) **OR** (toxicity) **OR** (health effects) **OR** (neurotoxin*) **OR** (paralysis))<br>*Query Search Science Direct:* ((tetrodotoxin) **OR** (anhydrotetrodotoxin) **OR** (4-epitetrodotoxin) **OR** (tetrodonic acid)) **AND** ((toxic) **OR** (toxicity) **OR** (health AND effects) **OR** (neurotoxin))
**Date of search Objective 1**	PubMed Search: date **01/10/2024**, at **12:42** A.M.<br>Scopus Search: date **01/10/2024**, at **13:45** A.M.<br>Science Direct Search: date **01/10/2024** at **10.45** A.M.
**Scientist involved**	Varini Carlo
**Number of records (PubMed)**	4321
**Number of records (Scopus)**	1226
**Number of records (Science Direct)**	4802
**Total N° retrieved**	12,741
**Number of duplicates removed**	2378
**Number undergoing primary screening – titles/abstracts**	10,363
**Number undergoing secondary screening**	360

**Number eligible for data abstraction**	17 plus 2 with supporting information on humans
**Search terms Objective 2**	Query Search PubMed: *(tetrodotoxin** ***OR*** *TTX)* ***AND*** *(presence** ***OR*** *occur** ***OR*** *concentration** ***OR*** *level** ***OR*** *bloom** ***OR*** *accumulation** ***OR*** *field** ***OR*** *laboratory*))* ***AND*** *(food** ***OR*** *fish** ***OR*** *shellfish** ***OR*** *crustacean** ***OR*** *mussel** ***OR*** *mollusk** ***OR*** *aquatic** ***OR*** *prawn** ***OR*** *seafood*) Filters: from 2017 - 2024*<br><br>Query Search Scopus: *TITLE-ABS-KEY ((tetrodotoxin** ***OR*** *ttx)* ***AND*** *(presence** ***OR*** *occur** ***OR*** **concentration** ***OR*** *level** ***OR*** *bloom** ***OR*** **accumulation** ***OR*** *field** ***OR*** *laboratory*)* ***AND*** *(food** ***OR*** *fish** ***OR*** *shellfish** ***OR*** *crustacean** ***OR*** *mussel** ***OR*** *mollusk** ***OR*** *aquatic** ***OR*** *prawn** ***OR*** *seafood*))*<br><br>Query Search Web of Science : *(((TS=(tetrodotoxin** ***OR*** *ttx))* ***AND*** *TS=(presence** ***OR*** *occur** ***OR*** **concentration** ***OR*** *level** ***OR*** *bloom** ***OR*** **accumulation** ***OR*** *field** ***OR*** *laboratory*)))* ***AND*** *TS=(food** ***OR*** *fish** ***OR*** *shellfish** ***OR*** *crustacean** ***OR*** *mussel** ***OR*** *mollusk** ***OR*** *aquatic** ***OR*** *prawn** ***OR*** *seafood*)*
**Date of search Objective 2**	24/07/2024
**Scientist involved**	Varini Carlo, Scardala Simona, Pietro Antonelli
**Number of records (PubMed)**	126
**Number of records (Scopus)**	140
**Number of records (Web of Science)**	141
**Total N° retrieved**	407
**Number of duplicates removed**	171
**Number undergoing primary screening – titles/abstracts**	236
**Number undergoing secondary screening**	22 review + 7 records
**Number eligible for data abstraction**	7
**Search terms Objective 3**	Query Search PubMed: **(tetrodotoxin* OR TTX) AND (presence* OR occur* OR concentration* OR level* OR accumulation* OR field* OR laboratory*) AND (pufferfish* OR lagocephalus OR tetraodontidae) AND (mediterranean OR thyrrenian OR libyan OR levantine OR aegean OR ionian OR balearic OR adriatic OR ligurian OR alboran)** Sort by: **Most Recent**<br><br>Query Search Scopus: *TITLE-ABS-KEY ((tetrodotoxin** ***OR*** *ttx)* ***AND*** *(presence** OR occur* OR concentration* OR

	level* OR accumulation* OR field* OR laboratory*) AND (pufferfish* OR lagocephalus OR tetraodontidae) AND (mediterranean OR thyrrenian OR libyan OR levantine OR aegean OR ionian OR balearic OR adriatic OR ligurian OR alboran)<br><br>Query Search Web of Science : *(TS=(tetrodotoxin* OR ttx) AND TS= (presence** OR occur* OR concentration* OR level* OR accumulation* OR field* OR laboratory*) AND TS=(pufferfish* OR lagocephalus OR tetraodontidae) AND TS=(mediterranean OR thyrrenian OR libyan OR levantine OR aegean OR ionian OR balearic OR adriatic OR ligurian OR alboran))
**Date of search Objective 3**	20/11/2024
**Scientist involved**	Varini Carlo, Scardala Simona
**Number of records (PubMed)**	21
**Number of records (Scopus)**	36
**Number of records (Web of Science)**	36
**Total N° retrieved**	93
**Number of duplicates removed**	54
**Number undergoing primary screening – titles/abstracts**	39
**Number undergoing secondary screening**	2 review + 14 records
**Number eligible for data abstraction**	14

Table S2 Data from FoodEx2. Only countries with a significant 95th percentiles of Acute Food Consumption Grams (g) in a single day – Consuming days only have been reported. The name of the surveys has been reported as well, while the exposure hierarchy from L5 to L7 have not been reported since they were the same as exposure hierarchy L4

Survey's country	Survey start year	Survey name	Population Group (L2)	Exposure hierarchy (L1)	Exposure hierarchy (L2)	Exposure hierarchy (L3)	Exposure hierarchy (L4)	Number of consuming days	% consuming days	Mean	Standard Deviation	Median	95th percentile
**France**	2007	Individual and national study on food consumption 2	Adults	Fish, seafood, amphibians, reptiles and invertebrates	Molluscs	Oysters	Oysters	122	0.8%	70.96	45.65	58.50	134.55
**Italy**	2005	Italian National Food Consumption Survey INRAN-SCAI 2005-06	Adults	Fish, seafood, amphibians, reptiles and invertebrates	Molluscs	Clams, cockles, arkshells	Clams	135	1.9%	53.21	37.63	38.50	135.53
**Italy**	2005	Italian National Food Consumption Survey INRAN-SCAI 2005-06	Adults	Fish, seafood, amphibians, reptiles and invertebrates	Molluscs	Mussels	Blue mussel	112	1.6%	52.96	67.14	38.50	161.98
**Portugal**	2015	National Food, Nutrition and Physical Activity Survey of the Portuguese general population	Adults	Fish, seafood, amphibians, reptiles and invertebrates	Molluscs	Clams, cockles, arkshells	Clams	131	2.0%	29.83	25.65	23.09	92.49
**Spain**	2009	Spanish Agency for Food Safety (AESAN) - FIAB Survey	Adults	Fish, seafood, amphibians, reptiles and invertebrates	Molluscs	Clams, cockles, arkshells	Clams	197	7.2%	12.87	11.92	9.75	49.00
**Sweden**	2016	RIKSMATEN ADOLESCENTS 2016	Adolescents	Fish, seafood, amphibians, reptiles and invertebrates	Molluscs	Mussels	Blue mussel	229	4.0%	5.00	7.44	1.97	18.67
